# Multiple Roles of SARS-CoV-2 N Protein Facilitated by Proteoform-Specific Interactions with RNA, Host Proteins, and Convalescent Antibodies

**DOI:** 10.1021/jacsau.1c00139

**Published:** 2021-06-15

**Authors:** Corinne A. Lutomski, Tarick J. El-Baba, Jani R. Bolla, Carol V. Robinson

**Affiliations:** † Physical and Theoretical Chemistry Laboratory, University of Oxford, South Parks Road, OX13QZ Oxford, U.K.

**Keywords:** mass spectrometry, protein, SARS-CoV-2, COVID-19, coronavirus, virus, nucleocapsid

## Abstract

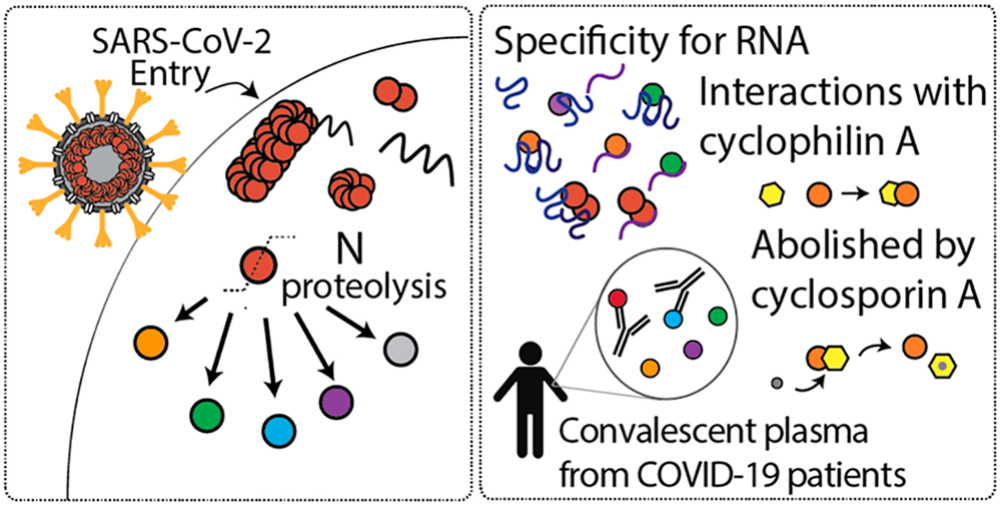

The SARS-CoV-2 nucleocapsid (N) protein is a highly immunogenic viral protein that plays essential roles in replication and virion assembly. Here, using native mass spectrometry, we show that dimers are the functional unit of ribonucleoprotein assembly and that N protein binds RNA with a preference for GGG motifs, a common motif in coronavirus packaging signals. Unexpectedly, proteolytic processing of N protein resulted in the formation of additional proteoforms. The N-terminal proteoforms bind RNA, with the same preference for GGG motifs, and bind to cyclophilin A, an interaction which can be abolished by approved immunosuppressant cyclosporin A. Furthermore, N proteoforms showed significantly different interactions with IgM, IgG, and IgA antibodies from convalescent plasma. Notably, the C-terminal proteoform exhibited a heightened interaction with convalescent antibodies, suggesting the antigenic epitope is localized to the C-terminus. Overall, the different interactions of N proteoforms highlight potential avenues for therapeutic intervention and identify a stable and immunogenic proteoform as a possible candidate for immune-directed therapies.

Severe acute respiratory syndrome coronavirus 2 (SARS-CoV-2) is the etiological agent of coronavirus disease 2019 (COVID-19), which reached pandemic status in fewer than 3 months following its discovery. As of March 2021, there were >100 million infected and more than 2.5 million deaths.^1^ SARS-CoV-2 packages a large RNA genome of ∼30 kb, which encodes for 25 nonstructural and 4 structural proteins (spike, nucleocapsid, membrane, and envelope proteins). The structural and genetic makeup of SARS-CoV-2 is highly homologous to SARS-CoV, the virus responsible for the 2002 SARS pandemic.^2^ Despite striking similarities between SARS-CoV and SARS-CoV-2, the unique biological and molecular features that have contributed to increased infectivity of SARS-CoV-2 are not well-understood.

Much attention has focused on the spike glycoprotein trimer, as it is the largest structural protein that plays a critical role in virus attachment to host cells.^3^ The nucleocapsid (N) protein, on the other hand, is one of the most abundant viral proteins; hundreds of copies of N make up the viral core that encapsulates the genomic RNA. N protein consists of two structured domains separated by a long flexible linker, giving rise to a high degree of conformational freedom. The separated domains allow the N protein to carry out many functions in the viral lifecycle such as RNA replication/transcription, virion assembly, and immune system interference.^4^ Interestingly, the nucleocapsid and spike proteins are the main immunogens in COVID-19 patients.^5^ Quantitative measurements of plasma or serum from SARS-CoV-2 patients found that the adaptive immune response to the N protein is more sensitive than to the spike protein, making it a better indicator of early disease.^6^ Antibodies against N protein are sensitive targets for COVID-19 diagnostics; however, the modes of action of anti-N antibodies in immunity and viral clearance are not well-understood.^7^

Efforts to elucidate the structure of N protein aim to understand how the individual domains interact with RNA and other proteins.^8,9^ However, N protein is highly sensitive to proteolysis^10^ due to the intrinsically disordered linker region, and therefore, the structure of the full-length nucleocapsid protein remains elusive.^11^ Proteolysis is a key strategy in viral proliferation^12^ and often begets a change in protein function. Understanding the unique biological features of SARS-CoV-2, and the ability to target specific viral processes, relies on an understanding of the sequence of interactions between viral and host proteins, inclusive of viral proteoforms.

Here, we present a comprehensive analysis of the SARS-CoV-2 N protein using native mass spectrometry (MS), top-down fragmentation, and bottom-up sequencing. We find that the full-length N protein undergoes proteolysis at highly specific sites to generate at least five unique proteoforms. We identify various stoichiometries of the proteoforms that are influenced by pH and evaluate the propensity for N proteoforms to bind different RNA sequences. We find that only the dimeric form of full-length N binds to RNA, suggesting it is the functional unit of ribonucleoprotein assembly. In addition, we show that an N-terminal proteoform directly interacts with cyclophilin A, a highly abundant cytosolic host protein implicated in viral replication. Moreover, cyclosporin A, an immunosuppressive drug, abolishes the interaction between N and cyclophilin A. Finally, using convalescent plasma from patients >6 months from initial COVID-19 diagnosis, we found that N proteoforms produced significantly different responses to IgM, IgG, and IgA antibodies. Notably, the C-terminal proteoform exhibited a heightened interaction with convalescent antibodies, suggesting the antigenic epitope is localized to the C-terminus. Our results contribute proteoform-specific information that may guide some of the many therapies against COVID-19 that are under investigation.

## Results and Discussion

### N Protein Undergoes Proteolysis in the Vicinity of the Linker Region

Nucleocapsid proteins of coronaviruses share similar topological organization^13^ and show high sequence homology among related coronaviruses.^14^ The N protein is characterized by two major structural domains, the RNA binding and oligomerization domains. Similar to other coronavirus nucleocapsid proteins, the two domains are separated by a long and flexible linker region thought to be devoid of secondary structure.^8^ An N-terminal arm and a C-terminal tail flank the RNA binding and oligomerization domains, respectively. We constructed plasmids consisting of the full-length nucleocapsid protein with a cleavable N-terminal purification tag and expressed the constructs in both *Escherichia coli* and human embryonic kidney (HEK) 293T cells.

For the *Escherichia coli* construct, we purified the protein and verified the cleavage of the affinity tag via SDS-PAGE (Figure S1). Before removal of the purification tag, three distinct protein bands were detected at ∼49, ∼38, and ∼28 kDa. Following removal of the tag, all three protein bands migrated by ∼3 kDa less, or the mass of the tag (Figure S1B). We confirmed that each band corresponded to N protein by in-gel trypsin digestion followed by LC–MS-based bottom-up proteomics. All three bands contained peptides from the N protein, resulting in 78.1, 49.9, and 43.2% sequence coverage for bands 1, 2, and 3, respectively. To determine the representation of protein domains in each gel band, we plotted the distribution of the peptides detected across the five protein domains (Figure S1C). As anticipated, we observe an unbiased distribution of peptides across all five domains for band 1, consistent with the expectation that tryptic peptides would be reasonably distributed across the full-length protein. Over 50% of the total peptides detected in bands 2 and 3 were localized to the RNA binding domain, suggesting that the proteins are predominantly N-terminal derivatives. However, in all three bands, peptides located in the 58-residue C-terminal domain were detected, indicating the purified protein is made up of a diverse mixture of N proteoforms^15^ and not biased to N-terminal species due to the location of the purification tag.

**Figure 1:**
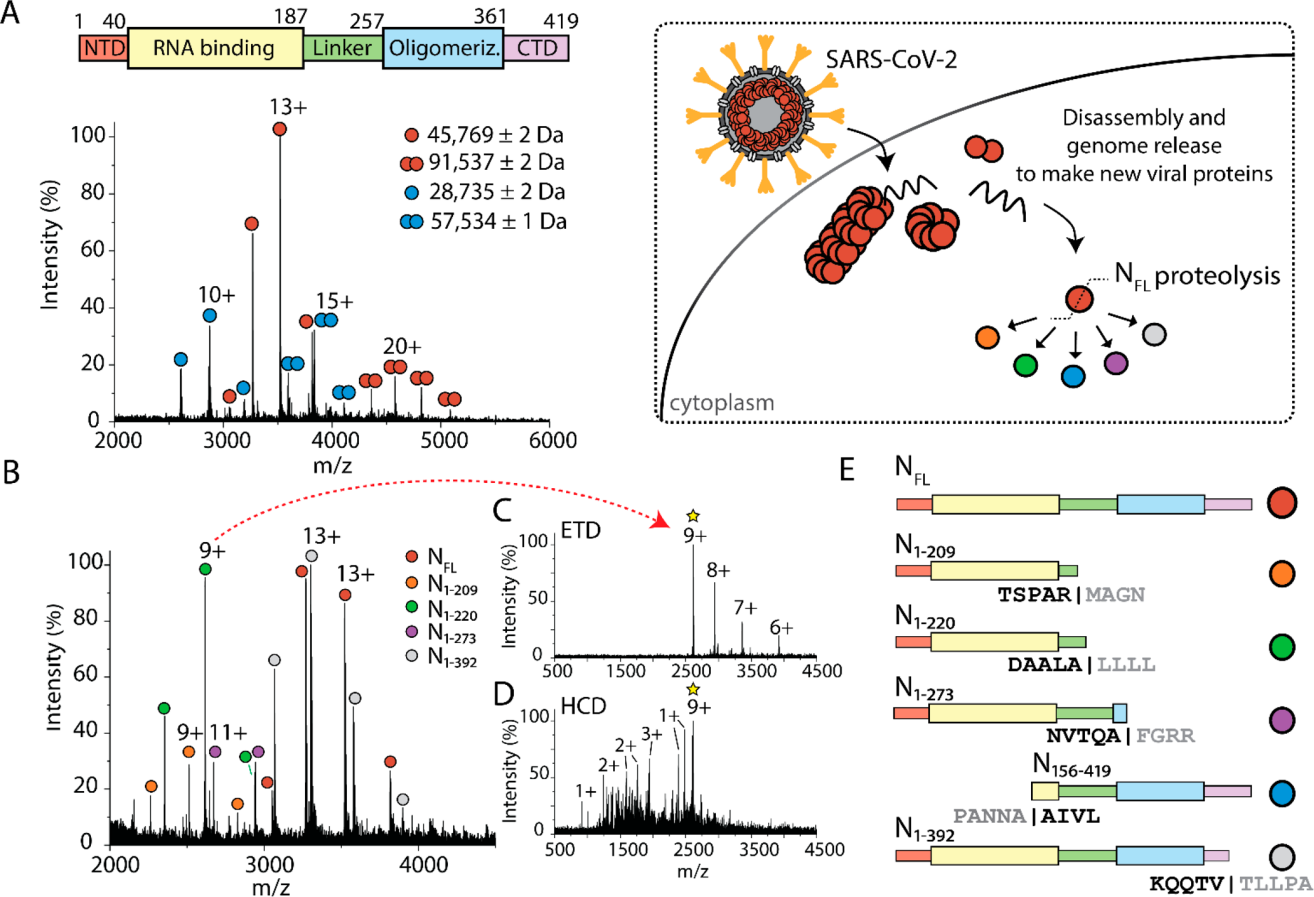
SARS-CoV-2 N protein exists as an ensemble of proteoforms. (A) Scheme depicting the full-length construct for expression in *E. coli* and native mass spectrum of the full-length N protein. Four charge state distributions correspond to monomers and dimers of full-length N protein (red circle, MW 45 769 Da) and a proteoform of N protein (blue circle, MW 28 735 Da). (B) Mass spectrum of N protein after several days at room temperature reveals the coexistence of five distinct charge state distributions corresponding to N proteoforms. The chemical composition of each proteoform was determined using top-down MS (C,D). (C) Charge-reduced mass spectrum resulting from electron-transfer dissociation (ETD) of the selected 9+ charge state at *m*/*z* 2616.79. (D) Mass spectrum of sequence ions for the same parent ion generated by higher-energy collision induced dissociation (HCD). (E) Scheme representing the composition of protein domains for the observed proteoforms as determined by top-down MS. Five distinct proteoforms are observed: N_1–209_, N_1–220_, N_1–273_, N_156–419_, and N_1–392_. The exact site of cleavage, including the five residues flanking either side of each cleavage site, is indicated below each construct.

We recorded a native mass spectrum to identify the stoichiometry of N protein from *E. coli* (Figure 1A) and observed two main charge state envelopes centered at 13+ and 20+. Deconvolution of the *m*/*z* signals provided experimental masses of 45 769 ± 1 and 91 537 ± 2 Da, respectively, which are in excellent agreement with the theoretical monomeric (45 769.83 Da) and dimeric (91 539.66 Da) masses of full-length N protein. A second distribution of monomers and dimers was observed and found to have deconvoluted masses of 28 735 ± 2 and 57 534 ± 1 Da, respectively.

Additional N proteoforms were observed immediately upon protein purification and continued to develop further over time (Figure S2). The mass spectrum evolved to reveal a series of peaks corresponding to five unique protein distributions (Figure 1B). The high baseline in this mass spectrum suggests the presence of additional proteolytic products; however, their low signal-to-noise precludes their characterization. Furthermore, it is likely that C-terminal proteoforms, containing the oligomerization domain, participate in the formation of higher-order oligomers that become insoluble and are therefore not detected at later time points. We instead sought to characterize the most stable and prominent proteoforms.

To determine the identity of each proteoform, we adapted a two-tiered tandem mass spectrometry approach^16^ to determine the intact mass and amino acid sequence for each series of peaks in the mixture. An individual peak in the mass spectrum was first isolated and subjected to charge reduction via electron-transfer dissociation (ETD) under conditions that do not result in the formation of fragments but instead produce a series of charge-reduced peaks (Figure 1C). The charge-reduced spectrum was necessary to confirm the assignment of the charge state series for each proteoform. The assigned charge states were then used to obtain deconvoluted masses of each proteoform present in solution (Table 1).

**Table 1:** Deconvoluted and Sequence Masses of N_FL_ and N Proteoforms

protein	deconvoluted mass^a^ ± s.d. (Da)	sequence mass (Da)
N_FL_	45 769 ± 2	45 769.83
N_FL_ dimer	91 537 ± 2	91 539.66
N_156–419_	28 735 ± 2	28 696.12
N_156–419_ dimer	57 534 ± 1	57 392.24
N_1–209_	22 611 ± 1	22 612.71
N_1–220_	23 540 ± 0.3	23 541.73
N_1–273_	29 402 ± 0.6	29 382.43
N_1–392_	42 922 ± 1	42 918.74

a Determined using at least three adjacent charge states.

We tentatively assigned the proteoforms based on their intact masses and then generated sequence ions to confirm our assignment. Individual proteoforms were subjected to fragmentation by higher-energy collision induced dissociation (HCD), which accelerates the isolated ions into an inert gas to induce fragmentation along the amide backbone. The fragmentation products were then used to determine the molecular composition and to localize the exact site of cleavage. The HCD spectrum results in a series of singly, doubly, and triply charged sequence ions exemplified by the fragmentation of the 9+ charge state at *m*/*z* 2616.79 (Figure 1D). Fragmentation of intact proteins under native conditions is expected to yield 3–10% sequence coverage at the termini,^17^ and the propensity for fragmentation differs depending on several criteria (e.g., mass, charge, composition, structure) for natively folded proteins.^18^ Here, we achieved ∼4% sequence coverage of the proteoforms by native top-down MS (Tables S1–S5). Fragmentation at the termini was complementary to our goal—to confirm the chemical identity of the distinct proteoforms of N protein.

To confirm that N proteoforms exist in a human-derived cell line, we expressed N protein in HEK 293T cells. We assessed N protein expression by Western blot using a monoclonal antibody raised against the highly homologous SARS-CoV N protein with cross-reactivity to SARS-CoV-2 N protein (Figure S3). Analysis of the whole cell lysate of cells stably expressing N protein produced mainly full-length N protein with evidence of lower-molecular-weight proteoforms (Figure S3A). Notably, when N is coexpressed with the membrane and envelope viral structural proteins, N proteoforms are generated in high abundance (Figure S3B). The enhanced cleavage of the full-length N protein in the presence of known interactors^19^ suggests that N proteoforms are primed for viral protein–protein interactions.

Having demonstrated that a number of proteoforms of N are generated in *E. coli* and HEK 293 cells, we continued our studies using protein derived from *E. coli* to characterize the proteoforms generated as a consequence of N protein primary structure. We reasoned that N from human cells is subject to additional post-translational modifications depending on the presence of co-interactors and the biological state of the cell during an infection.^20^ Considering the proteoforms identified in *E. coli*, we note that three result from cleavage after alanine (residue pairs A|L, A|F, and A|A), one results from cleavage following an arginine (R|M), and one results from cleavage in the C-terminal tail following a valine (V|T), (Figure 1E). Proteoforms N_1–209_ and N_1–220_ contain primarily the RNA binding domain, as they cleave within the flexible linker region. The major component of N_1–273_ is also the RNA binding domain but, in this case, is followed by the linker region and a small portion of the oligomerization domain. Conversely, N_156–419_ comprises mainly the oligomerization domain while still retaining a small portion of the RNA binding domain.

Although we find no sequence similarity in the residues that flank each cleavage site, a commonality is that cleavage occurs immediately adjacent to a hydrophobic residue. The specificity of cleavage at the residues identified here, despite no common motif, suggests that there may be a structural or conformational component directing proteolysis; however, the exact mechanism is unclear. While the exact chemical identity of N proteoforms implicated in SARS-CoV-2 infection may vary, we suggest that proteolytic processing of N protein, which liberates the two major domains, produces functionally relevant proteoforms that are important antiviral targets.

### Oligomeric States of N Proteoforms Are Influenced by pH

To better understand the role of the individual proteoforms, we expressed and purified four of the proteoforms identified by top-down MS. As almost all biological processes are influenced by pH, we recorded native mass spectra of N_1–209_, N_1–220_, N_1–273_, and N_156–419_ from solutions at different pH (Figures S4–S8) to mimic the pH of different intracellular environments that N might encounter during viral infection.^21^ Mass spectra for N_1–209_ and N_1–220_ recorded at pH 5.0, 7.4, and 8.0 reveal highly abundant charge state series centered at 9+ and a low-abundance distribution of signals centered at the 13+ charge state corresponding to monomers and dimers, respectively. The mass spectra for N_1–273_ show that it is predominantly monomeric with no significant change in charge state distribution or oligomeric state across the range of pH values tested. However, we observe two low-abundance charge state series corresponding to N_1–209_ and N_1–220_, suggesting that N_1–273_ continues to undergo cleavage at the previously mapped residues (Figure S6).

In contrast to the N-terminal proteoforms, mass spectra for N_156–419_ reveal multiple charge state distributions at pH 5.0, 7.4, and 8.0 (Figure S7). At pH 8, the mass spectrum reveals three charge state distributions centered at 10+, 18+, and 27+ with average masses corresponding to monomers, trimers, and a low population of hexamers (Table S6). At pH 7.4, monomers, dimers, and trimers persist. At the lowest pH (pH 5.0) N_156–419_ is exclusively trimeric. Finally, the mass spectra for full-length N at pH 5.0 and 8.0 reveal broadened and featureless peaks suggesting that N_FL_ is likely aggregated (Figure S8). A low-abundance series of highly charged peaks centered at 18+ at pH 8.0 indicates some protein unfolding. Overall, we conclude that N_1–209_, N_1–220_, and N_1–273_ do not undergo significant pH-dependent changes in oligomeric state, while the C-terminal proteoform is highly sensitive with trimers predominating under both high- and low-pH conditions.

### RNA Sequence Influences Binding Stoichiometry

RNA binding and ribonucleoprotein complex formation is the primary function of coronavirus N proteins. The N protein binds nucleic acid nonspecifically;^22^ however, the production of infectious virions relies on N protein forming specific interactions with viral RNA among an abundance of different cellular RNA species. With knowledge of the stoichiometries of N proteoforms, we sought to determine the propensity for N to bind specific RNA sequences. We created single-stranded RNA oligonucleotides consisting of 20 nucleotides of repeating sequences (4 × −GAUGG, 4 × −GAGAA). Considering the promiscuity of N protein, we chose sequences shown to interact with the human immunodeficiency virus (HIV) polyprotein (which includes a nucleocapsid domain) at the different stages of virus assembly^23^ and hypothesized that N proteins of different oligomeric states would exhibit similar bias toward artificial RNA motifs.

**Figure 2:**
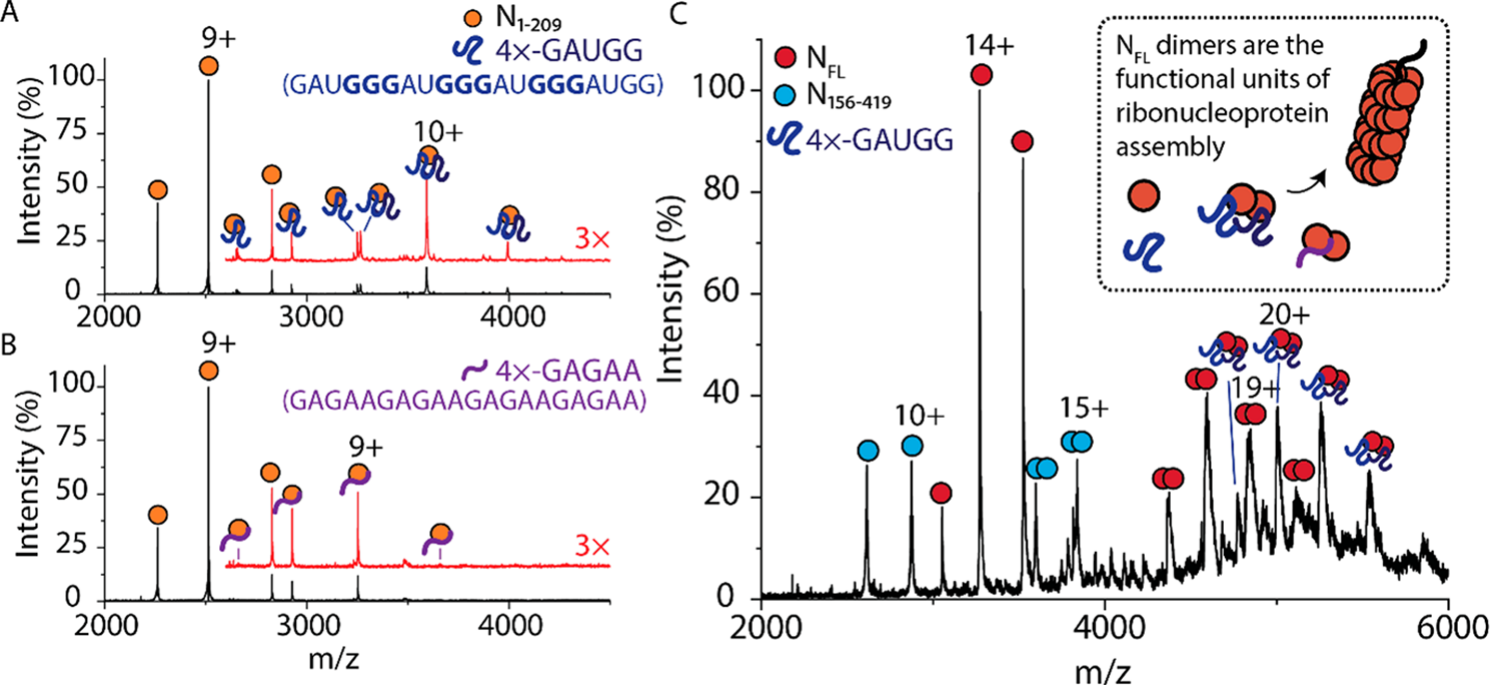
RNA sequence influences the binding stoichiometry to N protein. (A) Mass spectrum of N_1–209_ after incubation with 4 × −GAUGG RNA oligonucleotides in a molar ratio of 1:4 RNA:protein. Two additional charge state distributions are observed that correspond to one and two RNA oligonucleotides bound to N_1–209_. The mass spectrum at *m*/*z* > 2700 was magnified 3× and offset for clarity (red trace). (B) Mass spectrum of N_1–209_ after incubation with 4 × −GAGAA RNA oligonucleotides in a molar ratio of 1:4. One additional charge state distribution is observed that corresponds to one RNA oligonucleotide bound to N_1–209_. The mass spectrum at *m*/*z* > 2700 was magnified 3× and offset for clarity (red trace). (C) Mass spectrum of N_FL_ after incubation with 4 × −GAUGG RNA oligonucleotides in a molar ratio of 1:4. Monomers and dimers of N_FL_ (red circles) and N_156–419_ (blue circles) are observed. An additional peak series between 4300 and 5600 *m*/*z* corresponds to two 4 × −GAUGG RNA oligonucleotides bound to N_FL_ dimer. The scheme in the inset of (C) depicts N_FL_ dimer bound to RNA as the functional unit of the ribonucleoprotein assembly.

We incubated N proteoforms and RNA oligonucleotides at a molar ratio of 4:1 protein/RNA and recorded native mass spectra for all N protein–RNA complexes. The mass spectrum for N_1–209_ bound to 4 × −GAUGG (Figure 2A) reveals three charge state distributions with masses that correspond to the apo N_1–209_ monomer and N_1–209_ bound to one and two 4 × −GAUGG RNA oligonucleotides. The charge state distribution corresponding to N_1–209_ bound to two 4 × −GAUGG RNA predominates over the single RNA-bound protein. Conducting the same experiment with a different oligonucleotide (4 × −GAGAA) reveals only one additional charge state distribution for N_1–209_ bound to one oligonucleotide (Figure 2B). Similar RNA binding stoichiometries are observed for N_1–220_ and N_1–273_; the mass spectra for N_1–220_ and N_1–273_ reveal distributions corresponding to the binding of one and two 4 × −GAUGG oligonucleotides. Only one additional distribution is observed for N_1–220_ or N_1–273_ incubated with a 4 × −GAGAA oligonucleotide, which corresponds to one oligonucleotide bound (Figures S9–10, Table S7), confirming the preference for the 4 × −GAUGG sequence for all N-terminal constructs.

To examine if this preference was also observed for full-length N, we incubated the protein with a 4 × −GAUGG oligonucleotide. A series of peaks was identified corresponding to the N protein dimer bound to two 4 × −GAUGG oligonucleotides (Figure 2C). Similarly, in the presence of the 4 × −GAGAA RNA oligonucleotide, an additional RNA-bound distribution is observed; however, the deconvoluted mass indicates that only one 4 × −GAGAA oligonucleotide is bound to the N_FL_ dimer (Figure S11, Table S7). No RNA binding to the monomeric form of N_FL_ is observed, regardless of oligonucleotide sequence. Considering the different properties of the two RNA oligonucleotides, the 4 × −GAUGG oligonucleotide contains three GGG motifs, which form short stem loops and are known to contribute additively to the efficiency of genome packaging in related viruses.^24^ Furthermore, selective RNA packaging has been described as a feature of innate immune response evasion.^25^ Our results emphasize that RNA sequence, and likely the secondary structure, is important for interactions with the N-protein. Notably, only the N_FL_ dimer binds RNA, suggesting that the dimer is the functional unit of the SARS-CoV-2 ribonucleoprotein assembly. The preference for the RNA sequence known to form stem loops also has implications in efficient genome packaging and likely contributes to an optimized packing density in the intact virion.

### N_1–209_ Interacts Directly with Cyclophilin A

**Figure 3:**
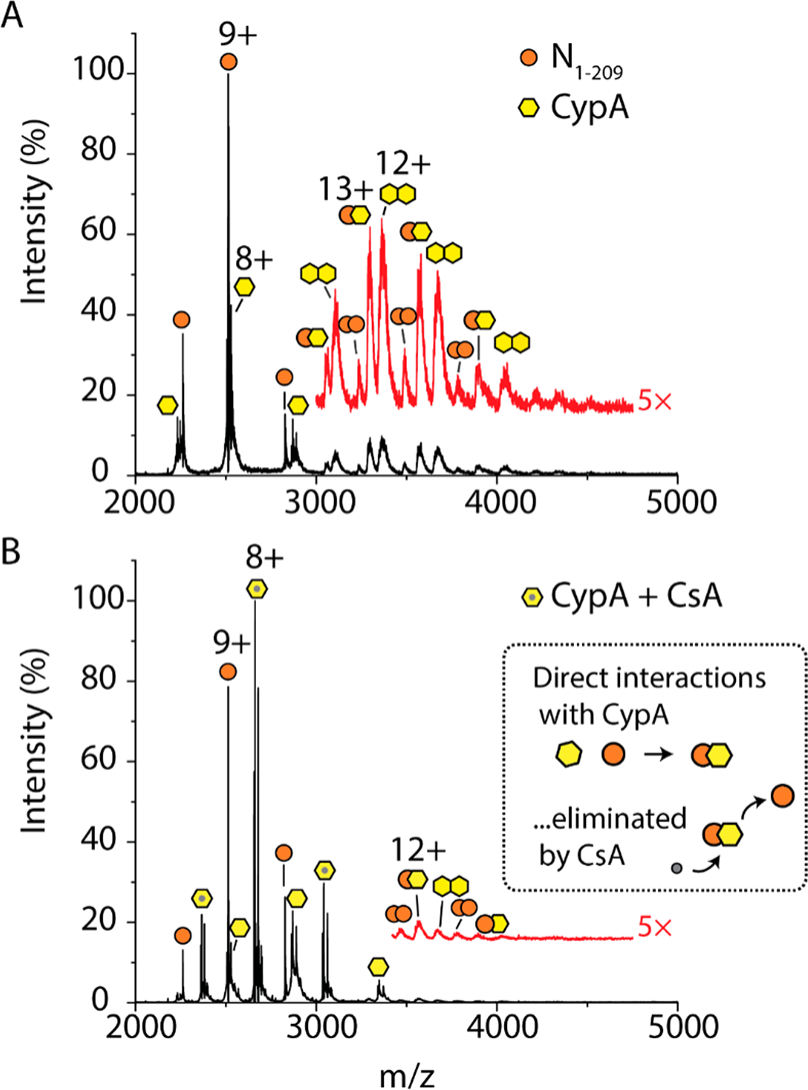
N proteoforms directly interact with cyclophilin A. (A) Mass spectrum of N_1–209_ after incubation with cyclophilin A (CypA) in a 1:1 molar ratio. The mass spectrum at *m*/*z* > 3000 was magnified 5× and offset for clarity (red trace). Three charge state distributions that correspond to a low population of homodimers of N_1–209_, homodimers of CypA, and heterodimers of N_1–209_–CypA. (B) Mass spectrum of N_1–209_ after incubation with CypA and cyclosporin A (CsA) in a molar ratio of 1:1:2. CypA preferentially binds CsA as demonstrated by the charge state distribution centered at 8+, which corresponds to the CypA–CsA complex. The mass spectrum at *m*/*z* > 3500 was magnified 5× and offset (red trace) to highlight the exceedingly low abundance of N_1–209_–CypA heterodimers that persist after CsA treatment. The scheme (inset of B) depicts CsA competitively binding to CypA and abolishing the N_1–209_–CypA interaction.

Cyclophilin A (CypA), a highly abundant immunophilin found in host cells, has been implicated in the replication cycle of coronaviruses^26^ and is known to aid in assembly of other viruses.^27^ We sought to determine if CypA could play a role in SARS-CoV-2 infection through monitoring direct interactions of CypA with N proteoforms. We incubated N_1–209_ and CypA in a 1:1 molar ratio and used native mass spectrometry to measure possible interactions. The mass spectrum reveals charge state distributions that correspond to monomeric N_1–209_ and CypA and three distinct charge state distributions at *m*/*z* > 3000 (Figure 3A). The three higher-*m*/*z* distributions correspond to (i) heterodimers of N_1–209_ and CypA, (ii) homodimers of CypA, and (iii) a low population of homodimers of N_1–209_ (Table 2). Notably, we do not observe evidence of CypA interacting with N_1–220_, N_1–273_, nor N_FL_ under the same conditions (Figure S12–15).

**Table 2:** Deconvoluted and Sequence Masses for N Proteoform Complexes

protein/complex	deconvoluted mass^a^ ± s.d. (Da)	expected mass (Da)
4 × −GAUGG RNA	--	6622.00
4 × −GAGAA RNA	--	6650.20
N_1–209_ + one 4 × −GAUGG RNA	29 233 ± 0.4	29 234.71
N_1–209_ + two 4 × −GAUGG RNA	35 917 ± 10	35 856.71
N_1–209_ + one 4 × −GAGAA RNA	29 262 ± 1	29 262.91
N_FL_ dimer + two 4 × −GAUGG RNA	105 131 ± 60	104 783.66
cyclophilin A (CypA)	20 084 ± 0.4	20 175.82
	20 220 ± 0.8	
CypA dimers	40 373 ± 62	40 351.64
N_1–209_ + CypA	42 867 ± 15	42 696.71
N_1–209_ dimers	45 356 ± 15	45 225.42
cyclosporin A	1202.85 ± 0	1202.63
CypA + CsA	21 287 ± 0.5	21 286.63
mAb (monomer)	137 990 ± 125	--
	145 193 ± 109	
N_FL_ + mAb	183 931 ± 102	183 759
		190 962
2N_FL_ + mAb	229 984 ± 36	229 528
		236 731

a Determined using at least three adjacent charge states.

To determine if the interaction between N_1–209_ and CypA could be inhibited by an approved immunosuppressant cyclosporin A (CsA), we incubated the N_1–209_:CypA complex with a 2-fold molar excess of the drug (Figure 3B). The mass spectrum reveals three abundant charge state distributions: (i) a distribution centered at 9+ corresponding to N_1–209_ monomers, (ii) a highly abundant distribution centered at a charge state of 8+ that corresponds to CypA bound to CsA, and (iii) a low-abundance distribution that corresponds to monomeric CypA (Table 2). Very low-abundance distributions of N_1–209_ homodimers, CypA homodimers, and N_1–209_-CypA heterodimers are barely detected following magnification >3000 *m*/*z*. Therefore, we can conclude that the 1:1 interaction between CypA and N_1–209_ can be inhibited by CsA binding to CypA.

### Antigenic Regions of N Are Located at the C-Terminus

**Figure 4:**
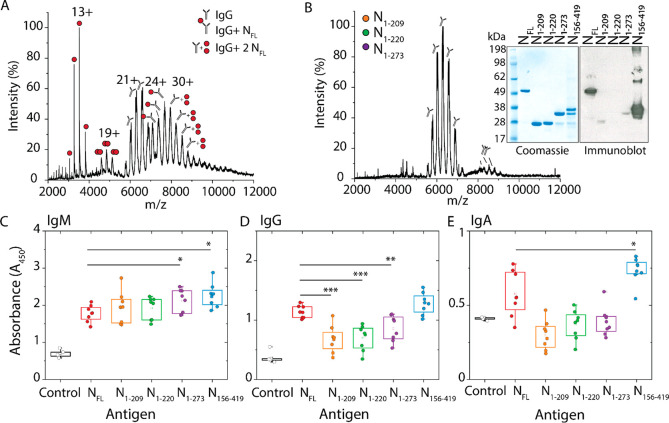
N proteoforms show significantly different interactions with antibodies. (A) Mass spectrum of N_FL_ after incubation with a monoclonal antibody raised against the full-length N protein in a molar ratio of 1:1. We observe five charge state distributions that correspond to N_FL_ monomers (centered at 13+), N_FL_ dimers (centered at 19+), a monomeric antibody (centered at 21+), an antibody bound to one NFL (centered at 24+), and a population of antibodies bound to N_FL_ dimer (centered at 30+). (B) Mass spectrum of a mixture of N_1–209_, N_1–220_, and N_1–273_ incubated with a monoclonal antibody in a molar ratio of 1:1:1:1. Charge state distributions are observed for antibody monomers and dimers. No binding to the antibody is observed for N proteoforms. This result was confirmed by immunoblot (see inset). SDS-PAGE shows that N_FL_, N_1–209_, N_1–220_, N_1–273_, and N_156–419_ are detected in high abundance by Coomassie stain. The same proteins analyzed by immunoblot show that only N_FL_ and N_156–419_ are detected by the antibody. The box-and-whisker plots depict the antibody response for (C) IgM antibodies, (D) IgG antibodies, and (E) IgA antibodies from plasma from eight patients collected >6 months following initial COVID-19 diagnosis. The antibody response was determined using the absorbance following colorimetric detection of a sandwich ELISA where the immobilized antigen was N_FL_, N_1–209_, N_1–220_, N_1–273_, or N_156–419_. The squares represent the mean, the center line represents the median, and the box represents the first quartile (25–75%) of the distributed data. Asterisks represent statistically significant differences when compared to N_FL_; *p*-values denoted by asterisks are defined as * *p* < 0.05, ** *p* < 0.01, and *** *p* < 0.001.

Since N protein is detected by antibodies with higher sensitivity than any other structural proteins of SARS-CoV-2, we sought to determine if all N proteoforms were similarly recognized by immunoglobulins. We first incubated N_FL_ with a monoclonal antibody (mAb) raised against the full-length protein in molar ratios of 1:1 (Figure 4A). The mass spectrum reveals three distributions >6000 *m*/*z* with deconvoluted masses that correspond to the apo antibody as well as one and two N_FL_ proteins bound (Table 2, Figure S16). When the same mAb was incubated with three N-terminal proteoforms (N_1–209_, N_1–220_, and N_1–273_) we did not observe any mAb binding in the mass spectrum (Figure 4B). To validate these results with conventional methods, we turned to protein detection by Coomassie stain and immunoblotting (Figure 4B, inset). The Coomassie stain detected N_FL_ and all proteoforms in high abundance. In contrast, the immunoblot revealed detection of only N_FL_ and N_156–419_, including higher-order oligomers of N_156–419_, which were not detected by Coomassie staining or mass spectrometry. N_1–209_ and N_1–273_ are barely detectable by the mAb, and N_1–220_ completely evades mAb detection. This suggests that the binding epitope recognized by immunoglobulins is localized near the C-terminus of N protein. If this were the case, then we would expect to see a heightened antibody response to the C-terminal proteoform relative to other proteoforms in convalescent plasma from patients with COVID-19.

To test this hypothesis, we obtained convalescent plasma from eight patients >6 months after an initial diagnosis of COVID-19 and studied the antibody response to the N proteoforms characterized herein. The experiment was carried out using an enzyme-linked immunosorbent assay (ELISA) using all five proteoforms (N_FL_, N_1–209_, N_1–220_, N_1–273_, N_156–419_) as the antigen and considering all three antibody responses (IgM, IgG, and IgA). The plasma antibodies were “sandwiched” using an anti-IgM, anti-IgG, or anti-IgA detection antibody conjugated with horseradish peroxidase for colorimetric detection. The antibody response for all eight patients was measured as a function of the absorbance and displayed as box plots (Figure 4C–E). More than 6 months after an initial diagnosis of COVID-19, there are still detectable levels of IgM, IgG, and IgA antibodies present against all N proteoforms in convalescent plasma. IgM antibodies were detected in the highest abundance with a statistically significant increase in detection for antibodies against C-terminal proteoforms N_1–273_ and N_156–419_. Furthermore, when compared to N_FL_, the N-terminal proteoforms (N_1–209_, N_1–220_, N_1–273_) exhibited a significantly attenuated IgG response. Interestingly, antibody interactions with N_FL_ and N_156–419_ were not statistically different, suggesting that the antigenic site for IgG recognition is localized to the C-terminus of the N protein. Finally, serum IgA antibodies were detected for all N proteoforms and showed a heightened response to N_156–419._ These results allow us to conclude that while all three antibodies are present, IgM antibodies are the most prevalent for all N proteoforms and IgG antibodies exhibit significant preference for the C-terminal proteoform.

## Conclusion

**Figure 5:**
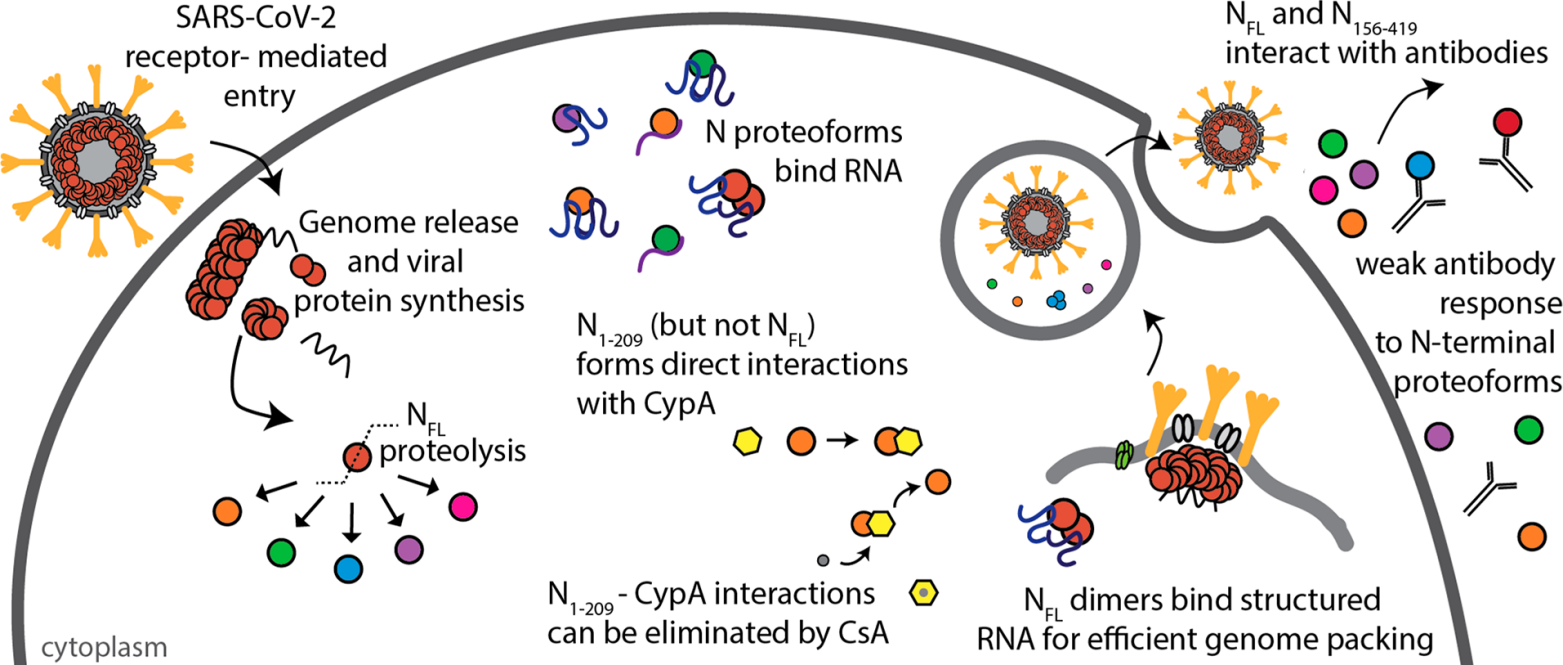
Scheme depicting features of SARS-CoV-2 N protein during infection. N protein undergoes proteolysis to produce N_1–209_, N_1–220_, N_1–273_, N_156–419_, and N_1–392_. N_FL_ and N proteoforms bind RNA with a preference for structured RNA, and N_FL_ dimers are likely functional unit of assembly in ribonucleoprotein complexes. Immunophilin CypA binds directly to N_1–209_ but not N_FL_, and the interaction can be inhibited through addition of cyclosporin A (CsA). N_156–419_ and N_FL_ interact with antibodies from convalescent plasma, while proteoforms N_1–209_, N_1–220_, and N_1–273_ fail to induce the same antibody response.

We present a comprehensive characterization of SARS-CoV-2 N protein and highlight molecular features that may influence the unique biology of SARS-CoV-2 infection (Figure 5). Specifically, we find that N protein undergoes proteolysis in the vicinity of the linker region, separating the two major domains (the RNA binding and oligomerization domains). We identify various stoichiometries of N proteoforms that are influenced by pH, explicitly N_156–419_, which forms oligomers under both high- and low-pH solution conditions. We also show that N proteoforms bind RNA with a preference for GGG motifs and present evidence for N_FL_ dimers being the functional unit of assembly in ribonucleoprotein complexes. Furthermore, we determined that immunophilin CypA binds directly to N_1–209_, an interaction that can be inhibited through addition of the immunosuppressant cyclosporin A. To test the immunogenicity of N proteoforms, we used a recombinant antibody and immunoblot techniques to demonstrate that the antigenic site of SARS-CoV-2 N protein resides toward the C-terminus. To test this in an *in vivo* scenario, we obtained convalescent plasma from eight patients >6 months after initial COVID-19 diagnosis. We discovered a heightened IgG and IgA response for the N_FL_ and N_156–419_ relative to N-terminal proteoforms N_1–209_, N_1–220_, and N_1–273._

The flexibility of the intrinsically disordered regions in proteins serves to provide conformational freedom for making favorable protein–protein interactions while making proteins highly susceptible to proteolytic cleavage. The nucleocapsid protein of SARS-CoV, responsible for the SARS pandemic of 2002, was shown to undergo similar proteolytic cleavage; however, the precise role of proteolysis in SARS-CoV N protein remains elusive.^28−30^ New approaches in proteomics have revealed proteolytic cleavage of multiple viral proteins during SARS-CoV-2 infection, including extensive proteolysis of the N protein.^31^ Furthermore, our results share striking similarities with the cleavage sites observed for both SARS-CoV and N protein from SARS-CoV-2-infected cells.^31^

One of the most critical steps of viral proliferation is the packaging of genomic material and its assembly into new virions. Genome packaging of RNA viruses is highly selective and depends on specific nucleotide sequences and complex structural elements called packaging signals (Psi).^32^ It has recently been demonstrated that HIV polyproteins have a stronger tendency to oligomerize in complex with Psi-RNA relative to non-Psi RNA.^33^ Our results indicate that N_FL_ and N proteoforms exhibit preference for RNA sequences that mimic key structural features of the genomic RNA.^34^ We anticipate that the more efficient binding of N protein dimers to RNA with GGG motifs underlies the selective packaging of genomic RNA in SARS-CoV-2. The disruption of structural features of viral RNA has been proven to inhibit replication in some viruses.^35^ In the case of SARS-CoV-2, disruption of preferred RNA structures, and therefore disruption of N protein–RNA interactions, presents a promising strategy to intervene in replication and the potential to develop live attenuated vaccines.

Our results demonstrate highly specific interactions between cyclophilin A and N_1–209_. Cyclophilin A has been found in mature virions of HIV and is known to play a key role in the replication of HIV, hepatitis B, and other coronaviruses^27^ Interestingly, the interaction between cyclophilin A and the HIV capsid protein is known to be conformation-dependent;^36^ we anticipate such parallels with SARS-CoV-2 and speculate that other N proteoforms do not interact with CypA because of a conformational change or restricted access to the interacting domains. Intervention of the specific interactions between N proteoforms and cyclophilin A could therefore prove beneficial as a component of treatment strategies.

Considering antibody responses, proteoforms N_1–209_, N_1–220_, and N_1–273_ show a diminished interaction with IgG antibodies in convalescent plasma when compared to the full-length protein. These proteoforms could thereby contribute to virtually unchecked virus proliferation, depending on their functional roles and the protective properties of anti-N IgG. By contrast, N_156–419_ resulted in a heightened IgM response and an IgG response that could not be differentiated from the full-length protein, suggesting that the antigenic site is localized to the C-terminus. This finding is in accord with epitope mapping of the homologous SARS-CoV N protein.^37−40^ The significant differences in IgM, IgG, and IgA detection highlight new avenues for therapies to direct immune responses and represent potential targets for next-generation vaccine development.^41^

Beyond the identification of potential vaccine targets, several additional therapeutic avenues are highlighted by this study. First, inhibition of the proteolysis reaction would prevent the formation of viral proteoforms that may be fine-tuned for various functions within the viral lifecycle. While the mechanism behind the proteolysis of N-protein is not yet understood, systematic mutation of cleavage sites defined here could lead to mechanistic and structural insights to enable small molecule screening of potential protease inhibitors. Knowledge of N-protein-structured RNA interactions could also aid the design of new therapeutics that would inhibit successful replication. However, since N protein oligomerizes in the absence of RNA,^42^*de novo* drug design of assembly inhibitors is complex, as it is necessary to consider the oligomerization propensity of the various proteoforms at the pH regimes encountered in the cellular environment. CsA and cyclosporine derivatives, such as Alisporivir, however are more straightforward to track and have become attractive candidates to treat COVID-19.^43^ Disruption of the cyclophilin A–N-proteoform interactions shown here provides a convenient means of screening potential inhibitors for hit-to-lead optimization. Given the likelihood that no one intervention is likely to ameliorate the complex symptoms of COVID-19 infection, our findings contribute proteoform-specific information that may guide some of the many therapies under investigation.

## Materials and Methods

### Ethics

Patients were recruited from the John Radcliffe Hospital in Oxford, United Kingdom, between March and May 2020 with written and informed consent. Participants were identified from hospitalization during the SARS-COV-2 pandemic and recruited into the Sepsis Immunomics and International Severe Acute Respiratory and Emerging Infection Consortium World Health Organization Clinical Characterization Protocol UK (IRAS260007 and IRAS126600). Patient samples were collected at least 28 days from the start of their symptoms. Ethical approval was given by the South Central–Oxford C Research Ethics Committee in England (reference: 13/SC/0149), Scotland A Research Ethics Committee (reference: 20/SS/0028), and World Health Organization Ethics Review Committee (RPC571 and RPC572l; 25 April 2013).

### Plasmid Construction and Cell Growth

A codon-optimized synthetic gene corresponding to the full-length nucleocapsid protein (Thermo GeneArt, Regensburg, Germany) was cloned into a modified pET28a vector using the In-Fusion cloning kit (Takara Bio Saint-Germain-en-Laye, France). The resulting plasmid encoded for an N-terminal His_6_ tag followed by thrombin and tobacco etch virus cleavage sequences upstream of the full-length nucleocapsid protein sequence. To generate the nucleocapsid proteoforms, the desired sequences pertaining to the truncated forms of the N protein were subcloned from the synthetic gene using polymerase chain reaction (Phusion polymerase, New England Biolabs, Hertfordshire, UK). All genes were cloned into the modified pet28 vector, and gene sequences were confirmed by Sanger sequencing.

Plasmids were transformed into BL21 (DE3) and streaked onto LB agar plates supplemented with 50 mg mL^–1^ of kanamycin. Several colonies were used to inoculate 100 mL of LB broth supplemented with kanamycin and grown at 37 °C overnight. Aliquots of 10 mL of the overnight precultures were used to inoculate 1 L of LB broth supplemented with kanamycin. Cell cultures were grown to OD_600_ ≈ 0.6 before protein expression was induced with 0.5 μg mL^–1^ of IPTG. Cells were grown for an additional 4 h at 37 °C before being harvested via centrifugation (5000*g*, 10 min, 4 °C). Cell pellets were flash frozen in liquid nitrogen and stored at −80 °C until use.

### Protein Purification

Cell pellets were resuspended in lysis buffer (25 mM Tris-HCl pH 8.0, 500 mM NaCl, 5 mM MgCl_2_, 5 mM β-mercaptoethanol, 5 mM imidazole, 10% v/v glycerol) containing EDTA-free protease inhibitor tablets (Roche). Cells were lysed by five passes through a microfluidizer (prechilled to 4 °C) at 20 000 psi. Cell debris was pelleted by centrifugation (20 000*g*, 20 min, 4 °C). The supernatant containing soluble nucleocapsid protein was passed through a 0.45 μm filter.

Supernatant was loaded onto a Ni-NTA column pre-equilibrated in loading buffer (25 mM Tris-HCl pH 8.0, 500 mM NaCl, 5 mM MgCl_2_, 5 mM β-mercaptoethanol, 20 mM imidazole, 10% v/v glycerol) and allowed to pass via gravity flow. To remove common contaminating proteins, a heat-treated BL21 (DE3) *E. coli* lysate in loading buffer containing 10 mM MgATP was passed over the immobilized nucleocapsid protein using a protocol by Rial and Ceccarelli.^44^ The resin was washed with 10 column volumes of wash buffer (25 mM Tris-HCl pH 8.0, 500 mM NaCl, 5 mM MgCl_2_, 5 mM β-mercaptoethanol, 80 mM imidazole, 10% v/v glycerol) and then eluted twice with 10 mL of elution buffer (25 mM Tris-HCl pH 8.0, 500 mM NaCl, 5 mM MgCl_2_, 5 mM β-mercaptoethanol, 400 mM imidazole, 10% v/v glycerol).

The eluted protein was mixed with TEV protease in a 100:1 (w/w) ratio and loaded into a 3 kDa MWCO dialysis cassette (Thermo Fisher Scientific, United Kingdom). Cleavage of the His_6_–thrombin–TEV tag was carried out overnight at 4 °C in lysis buffer. The cleaved tag and TEV protease were separated from the untagged protein using reverse immobilized metal affinity chromatography on a Ni-NTA column prepared in loading buffer. The flow-through containing the untagged protein was collected and concentrated in a 10k MWCO centrifugal filter before MS analysis. Protein concentration was determined using UV–vis spectroscopy by monitoring the absorbance at 280 nm with a theoretical extinction coefficient (ε ≈ 43 890 M^–1^ cm^–1^) determined using the Expasy Protparam tool.

### Native Mass Spectrometry

RNA oligonucleotides of repeating sequences (4 × −GAUGG, 4 × −GAGAA) were purchased from Integrated DNA Technologies. Recombinant human cyclophilin A (product ab86219) was purchased from Abcam (Cambridge, United Kingdom). Cyclosporine A purchased from Merck Life Science (Dorset, United Kingdom). N protein and all binding partners were buffer exchanged or diluted into 500 mM NH_4_OAc pH 5.0, 7.4, or 8.0. Buffer exchange was carried out using Zeba Spin Desalting Columns, 7K MWCO (Thermo Fisher Scientific, United Kingdom).

Measurements were carried out on a Q Exactive UHMR or Orbitrap Eclipse. The Q Exactive instrument was operated in the positive ion mode using the manufacturer’s recommended parameters for native MS. The instrument was operated at a resolving power of 12 500 (at *m*/*z* 200). An electrospray was generated by applying a slight (∼0.5 mbar) backing pressure to an in-house-prepared gold-coated electrospray capillary held at ∼1.2 kV relative to the instrument orifice (heated to ∼100 °C). The mass spectra were deconvoluted using in-house software (the Mass and Charge State Evaluation and Determination tool, available for download at http://benesch.chem.ox.ac.uk/resources.html).

An Eclipse Tribrid instrument was also used for native MS and top-down sequencing. The instrument was set to intact protein mode at a standard ion routing multipole pressure of 10 mTorr. Ion voltages were set to transmit and detect positive ions at a resolving power of 12 500 (at *m*/*z* 200). An electrospray voltage of ∼1.2 kV and backing pressure of ∼0.5 mbar were used for ion formation; desolvation was assisted using an instrument capillary temperature of ∼100 °C. To identify the accurate mass and sequence of each proteoform, we used a similar approach to that described by Huguet et al.^16^ Briefly, a desired signal was isolated using the ion trap (10 *m*/*z* isolation window, charge state set to 10) and subjected to (i) electron-transfer dissociation (ETD, 3 ms activation time, 1.0 × 10^6^ ETD reagent target) to generate a charge-reduced series for accurate intact mass determination and (ii) higher-energy collisional dissociation (HCD) using ∼30–50 V HCD collision energy to generate fragment ions.

Monoisotopic masses of fragment ions generated by HCD having a normalized intensity of 10% or higher were fed into Prosite Lite software.^45^ A series of candidate sequences that best matched the measured intact masses for each proteoform were generated. The monoisotopic fragment masses were matched to expected ions generated in silico based on the provided candidate sequence. Internal fragments (peptides resulting from multiple fragmentation events that include neither the C-terminus nor the N-terminus of the protein) were excluded from the analysis. Comparison of the statistical likelihood for each match compared to a series of candidate sequences (Tables S1–S5) localized the cleavage sites to those outlined in Figure 1E.

### Western Blot

Recombinant antibody generated from the full-length SARS-CoV-2 nucleocapsid protein (product ab272852) and antihuman secondary antibody were purchased from Abcam. An additional recombinant monoclonal antibody raised against the SARS-CoV nucleocapsid protein (Invitrogen, MA5-29982) with cross-reactivity to the SARS-CoV-2 N protein was used for detection of N protein from HEK 293T cells. Proteins were resolved on a 4–12% Bis-tris gel using SDS-PAGE and transferred to a PVDF membrane (pore size 0.45 μm). The membrane was blocked in 5% milk in TPBS for 1 h at RT, incubated with primary antibody in 1:2000 dilution into blocking buffer (also 1 h, RT) washed with TPBS, and incubated with secondary antibody (1:10 000) in blocking buffer (also 1 h, RT). The PVDF membrane was incubated with horseradish peroxidase chemiluminescent substrate (Pierce ECL Western Blotting Substrate, Thermo Scientific) before detection on photographic film and developed by an X-ray film processor (Xograph Compact X4).

### Enzyme-Linked Immunosorbent Assay

Recombinant antibody generated from the full-length SARS-CoV-2 nucleocapsid protein (product ab272852) and goat antihuman IgM (ab97210), goat antihuman IgG (ab97160), and goat antihuman IgA (ab8510) conjugated with horseradish peroxidase (HRP) were purchased from Abcam. Nickel-coated clear 96-well plates were purchased from Thermo Scientific (Thermo Fisher Scientific, United Kingdom). Each well was loaded with 5 μg of his-tagged N protein or N proteoform and incubated at room temperature for 1 h. Plates were washed three times with 200 μL of phosphate buffered saline (PBS) containing 0.05% Tween-20 (PBST). Patient plasma was diluted 10-fold with PBS, and 100 μL was added to each well. Negative controls (five replicates) were run on each plate to assess absorbance due to nonspecific binding of secondary antibodies. In the negative controls, five wells were coated with N_FL_ and incubated with PBS instead of convalescent plasma. Antibodies from patient plasma and the controls were incubated overnight at 4 °C. Plates were washed three times with PBST. Goat antihuman IgG, IgM, and IgA secondary antibodies were diluted 1:50 000 in PBS, 100 μL was added to each well, including the controls, and the plates were incubated at room temperature for 1 h. Plates were washed a further three times with PBST. Colorimetric detection was carried out using a TMB chromogenic substrate kit for HRP detection (Thermo Fisher Scientific, United Kingdom). The reaction was quenched after 5 min using 2 M sulfuric acid, resulting in a yellow color. Absorbance measurements were immediately carried out at 450 nm using a microplate reader (BMG Labtech, Aylesbury, United Kingdom).

## Supporting Information Available

Supporting Information includes detailed experimental methods; Figures S1–S16; Tables S1–S7 (PDF)

## Abbreviations

SARS-CoV-2: severe acute respiratory syndrome coronavirus 2
COVID-19: coronavirus disease 2019
MS: mass spectrometry
N: nucleocapsid
HEK: human embryonic kidney
HCD: higher-energy collision induced dissociation
ETD: electron-transfer dissociation
CypA: cyclophilin A
CsA: cyclosporin A
mAb: monoclonal antibody
ELISA: enzyme-linked immunosorbent assay
Psi: packaging signals

## References

[ref1] Values taken from https://coronavirus.jhu.edu.

[ref2] Zhu Z. X.; Lian X. H.; Su X. S.; Wu W. J.; Marraro G. A.; Zeng Y. M. From SARS and MERS to COVID-19: a brief summary and comparison of severe acute respiratory infections caused by three highly pathogenic human coronaviruses. Respir. Res. 2020, 21 (1), 224 10.1186/s12931-020-01479-w. 32854739 PMC7450684

[ref3] Wrapp D.; Wang N. S.; Corbett K. S.; Goldsmith J. A.; Hsieh C. L.; Abiona O.; Graham B. S.; McLellan J. S. Cryo-EM structure of the 2019-nCoV spike in the prefusion conformation. Science 2020, 367 (6483), 1260–1263. 10.1126/science.abb2507. 32075877 PMC7164637

[ref4] McBride R.; van Zyl M.; Fielding B. C. The Coronavirus Nucleocapsid Is a Multifunctional Protein. Viruses 2014, 6 (8), 2991–3018. 10.3390/v6082991. 25105276 PMC4147684

[ref5] Okba N. M. A.; Muller M. A.; Li W. T.; Wang C. Y.; GeurtsvanKessel C. H.; Corman V. M.; Lamers M. M.; Sikkema R. S.; de Bruin E.; Chandler F. D.; Yazdanpanah Y.; Le Hingrat Q.; Descamps D.; Houhou-Fidouh N.; Reusken C. B. E. M.; Bosch B. J.; Drosten C.; Koopmans M. P. G.; Haagmans B. L. Severe Acute Respiratory Syndrome Coronavirus 2-Specific Antibody Responses in Coronavirus Disease Patients. Emerging Infect. Dis. 2020, 26 (7), 1478–1488. 10.3201/eid2607.200841. PMC7323511 32267220

[ref6] Burbelo P. D.; Riedo F. X.; Morishima C.; Rawlings S.; Smith D.; Das S.; Strich J. R.; Chertow D. S.; Davey R. T.; Cohen J. I. Sensitivity in Detection of Antibodies to Nucleocapsid and Spike Proteins of Severe Acute Respiratory Syndrome Coronavirus 2 in Patients With Coronavirus Disease 2019. J. Infect. Dis. 2020, 222 (2), 206–213. 10.1093/infdis/jiaa273. 32427334 PMC7313936

[ref7] Batra M.; Tian R.; Zhang C.; Clarence E.; Sacher C. S.; Miranda J. N.; De La Fuente J. R. O.; Mathew M.; Green D.; Patel S.; Bastidas M. V. P.; Haddadi S.; Murthi M.; Gonzalez M. S.; Kambali S.; Santos K. H. M.; Asif H.; Modarresi F.; Faghihi M.; Mirsaeidi M. Role of IgG against N-protein of SARS-CoV2 in COVID19 clinical outcomes. Sci. Rep. 2021, 11 (1), 3455 10.1038/s41598-021-83108-0. 33568776 PMC7875990

[ref8] Ye Q. Z.; West A. M. V.; Silletti S.; Corbett K. D. Architecture and self-assembly of theSARS-CoV-2 nucleocapsid protein. Protein Sci. 2020, 29 (9), 1890–1901. 10.1002/pro.3909. 32654247 PMC7405475

[ref9] Zinzula L.; Basquin J.; Bohn S.; Beck F.; Klumpe S.; Pfeifer G.; Nagy I.; Bracher A.; Hartl F. U.; Baumeister W. High-resolution structure and biophysical characterization of the nucleocapsid phosphoprotein dimerization domain from the Covid-19 severe acute respiratory syndrome coronavirus 2. Biochem. Biophys. Res. Commun. 2021, 538, 54–62. 10.1016/j.bbrc.2020.09.131. 33039147 PMC7532810

[ref10] Garvin M. R.; Prates E. T.; Pavicic M.; Jones P.; Amos B. K.; Geiger A.; Shah M. B.; Streich J.; Felipe Machado Gazolla J. G.; Kainer D.; Cliff A.; Romero J.; Keith N.; Brown J. B.; Jacobson D. Potentially adaptive SARS-CoV-2 mutations discovered with novel spatiotemporal and explainable AI models. Genome Biol. 2020, 21 (1), 304 10.1186/s13059-020-02191-0. 33357233 PMC7756312

[ref11] Peng Y.; Du N.; Lei Y.; Dorje S.; Qi J.; Luo T.; Gao G. F.; Song H. Structures of the SARS-CoV-2 nucleocapsid and their perspectives for drug design. EMBO J. 2020, 39, e105938 10.15252/embj.2020105938. 32914439 PMC7560215

[ref12] Kemp G.; Webster A.; Russell W. C. Proteolysis is a key process in virus replication. Essays Biochem 1992, 27, 1–16. 1425597

[ref13] Chang C. K.; Sue S. C.; Yu T. H.; Hsieh C. M.; Tsai C. K.; Chiang Y. C.; Lee S. J.; Hsiao H. H.; Wu W. J.; Chang W. L.; Lin C. H.; Huang T. H. Modular organization of SARS coronavirus nucleocapsid protein. J. Biomed. Sci. 2006, 13 (1), 59–72. 10.1007/s11373-005-9035-9. 16228284 PMC7089556

[ref14] Tilocca B.; Soggiu A.; Sanguinetti M.; Musella V.; Britti D.; Bonizzi L.; Urbani A.; Roncada P. Comparative computational analysis of SARS-CoV-2 nucleocapsid protein epitopes in taxonomically related coronaviruses. Microbes Infect. 2020, 22 (4–5), 188–194. 10.1016/j.micinf.2020.04.002. 32302675 PMC7156246

[ref15] Smith L. M.; Kelleher N. L. Proteoform: a single term describing protein complexity. Nat. Methods 2013, 10, 186–187. 10.1038/nmeth.2369. 23443629 PMC4114032

[ref16] Huguet R.; Mullen C.; Srzentic K.; Greer J. B.; Fellers R. T.; Zabrouskov V.; Syka J. E. P.; Kelleher N. L.; Fornelli L. Proton Transfer Charge Reduction Enables High-Throughput Top-Down Analysis of Large Proteoforms. Anal. Chem. 2019, 91 (24), 15732–15739. 10.1021/acs.analchem.9b03925. 31714757 PMC7008508

[ref17] Ives A. N.; Su T. J. F.; Durbin K. R.; Early B. P.; dos Santos Seckler H.; Fellers R. T.; LeDuc R. D.; Schachner L. F.; Patrie S. M.; Kelleher N. L. Using 10,000 Fragment Ions to Inform Scoring in Native Top-down Proteomics. J. Am. Soc. Mass Spectrom. 2020, 31 (7), 1398–1409. 10.1021/jasms.0c00026. 32436704 PMC7539637

[ref18] Haverland N. A.; Skinner O. S.; Fellers R. T.; Tariq A. A.; Early B. P.; Leduc R. D.; Fornelli L.; Compton P. D.; Kelleher N. L. Defining Gas-Phase Fragmentation Propensities of Intact Proteins During Native Top-Down Mass Spectrometry. J. Am. Soc. Mass Spectrom. 2017, 28 (6), 1203–1215. 10.1007/s13361-017-1635-x. 28374312 PMC5452613

[ref19] Alsaadi E. A. J.; Jones I. M. Membrane binding proteins of coronaviruses. Future Virol. 2019, 14 (4), 275–286. 10.2217/fvl-2018-0144. 32201500 PMC7079996

[ref20] Gordon D. E.; Jang G. M.; Bouhaddou M.; Xu J. W.; Obernier K.; White K. M.; O’Meara M. J.; Rezelj V. V.; Guo J. F. Z.; Swaney D. L.; Tummino T. A.; Huttenhain R.; Kaake R. M.; Richards A. L.; Tutuncuoglu B.; Foussard H.; Batra J.; Haas K.; Modak M.; Kim M.; Haas P.; Polacco B. J.; Braberg H.; Fabius J. M.; Eckhardt M.; Soucheray M.; Bennett M. J.; Cakir M.; McGregor M. J.; Li Q. Y.; Meyer B.; Roesch F.; Vallet T.; Mac Kain A.; Miorin L.; Moreno E.; Naing Z. Z. C.; Zhou Y.; Peng S. M.; Shi Y.; Zhang Z. Y.; Shen W. Q.; Kirby I. T.; Melnyk J. E.; Chorba J. S.; Lou K. V.; Dai S. Z. A.; Barrio-Hernandez I.; Memon D.; Hernandez-Armenta C.; Lyu J.; Mathy C. J. P.; Perica T.; Pilla K. B.; Ganesan S. J.; Saltzberg D. J.; Rakesh R.; Liu X.; Rosenthal S. B.; Calviello L.; Venkataramanan S.; Liboy-Lugo J.; Lin Y. Z.; Huang X. P.; Liu Y. F.; Wankowicz S. A.; Bohn M.; Safari M.; Ugur F. S.; Koh C.; Savar N. S.; Tran Q. D.; Shengjuler D.; Fletcher S. J.; O’Neal M. C.; Cai Y. M.; Chang J. C. J.; Broadhurst D. J.; Klippsten S.; Sharp P. P.; Wenzell N. A.; Kuzuoglu-Ozturk D.; Wang H. Y.; Trenker R.; Young J. M.; Cavero D. A.; Hiatt J.; Roth T. L.; Rathore U.; Subramanian A.; Noack J.; Hubert M.; Stroud R. M.; Frankel A. D.; Rosenberg O. S.; Verba K. A.; Agard D. A.; Ott M.; Emerman M.; Jura N.; von Zastrow M.; Verdin E.; Ashworth A.; Schwartz O.; D’Enfert C.; Mukherjee S.; Jacobson M.; Malik H. S.; Fujimori D. G.; Ideker T.; Craik C. S.; Floor S. N.; Fraser J. S.; Gross J. D.; Sali A.; Roth B. L.; Ruggero D.; Taunton J.; Kortemme T.; Beltrao P.; Vignuzzi M.; Garcia-Sastre A.; Shokat K. M.; Shoichet B. K.; Krogan N. J. A SARS-CoV-2 protein interaction map reveals targets for drug repurposing. Nature 2020, 583 (7816), 459–468. 10.1038/s41586-020-2286-9. 32353859 PMC7431030

[ref21] Ghosh S.; Dellibovi-Ragheb T. A.; Kerviel A.; Pak E.; Qiu Q.; Fisher M.; Takvorian P. M.; Bleck C.; Hsu V. W.; Fehr A. R.; Perlman S.; Achar S. R.; Straus M. R.; Whittaker G. R.; de Haan C.; Kehrl J.; Altan-Bonnet G.; Altan-Bonnet N. β-Coronaviruses Use Lysosomes for Egress Instead of the Biosynthetic Secretory Pathway. Cell 2020, 183, 1520–1535. 10.1016/j.cell.2020.10.039. 33157038 PMC7590812

[ref22] Zeng W.; Liu G.; Ma H.; Zhao D.; Yang Y.; Liu M.; Mohammed A.; Zhao C.; Yang Y.; Xie J.; Ding C.; Ma X.; Weng J.; Gao Y.; He H.; Jin T. Biochemical characterization of SARS-CoV-2 nucleocapsid protein. Biochem. Biophys. Res. Commun. 2020, 527 (3), 618–623. 10.1016/j.bbrc.2020.04.136. 32416961 PMC7190499

[ref23] Tanwar H. S.; Khoo K. K.; Garvey M.; Waddington L.; Leis A.; Hijnen M.; Velkov T.; Dumsday G. J.; McKinstry W. J.; Mak J. The thermodynamics of Pr55(Gag)-RNA interaction regulate the assembly of HIV. PLoS Pathog. 2017, 13 (2), e1006221 10.1371/journal.ppat.1006221. 28222188 PMC5336307

[ref24] Kim D. Y.; Firth A. E.; Atasheva S.; Frolova E. I.; Frolov I. Conservation of a Packaging Signal and the Viral Genome RNA Packaging Mechanism in Alphavirus Evolution. Journal of Virology 2011, 85 (16), 8022–8036. 10.1128/JVI.00644-11. 21680508 PMC3147971

[ref25] Athmer J.; Fehr A. R.; Grunewald M. E.; Qu W.; Wheeler D. L.; Graepel K. W.; Channappanavar R.; Sekine A.; Aldabeeb D. S.; Gale M. Jr; Denison M. R.; Perlman S. Selective packaging in murine coronavirus promotes virulence by limiting type I interferon responses. mBio 2018, 9, e00272-18 10.1128/mBio.00272-18. 29717007 PMC5930304

[ref26] Watashi K.; Shimotohno K. Cyclophilin and Viruses: Cyclophilin as a Cofactor for Viral Infection and Possible Anti-Viral Target. Drug Target Insights 2007, 2, 117739280700200 10.1177/117739280700200017. PMC3155236 21901058

[ref27] Zhou D. J.; Mei Q.; Li J. T.; He H. Y. Cyclophilin A and viral infections. Biochem. Biophys. Res. Commun. 2012, 424 (4), 647–650. 10.1016/j.bbrc.2012.07.024. 22814107 PMC7092870

[ref28] Mark J.; Li X. G.; Cyr T.; Fournier S.; Jaentschke L.; Hefford M. A. SARS coronavirus: Unusual lability of the nucleocapsid protein. Biochem. Biophys. Res. Commun. 2008, 377 (2), 429–433. 10.1016/j.bbrc.2008.09.153. 18926799 PMC7092863

[ref29] Ying W. T.; Hao Y. W.; Zhang Y. J.; Peng W. M.; Qin E.; Cai Y.; Wei K. H.; Wang J.; Chang G. H.; Sun W.; Dai S. J.; Li X. H.; Zhu Y. P.; Li J. Q.; Wu S. F.; Guo L. H.; Dai J. Q.; Wang J. L.; Wan P.; Chen T. G.; Du C. J.; Li D.; Wan J.; Kuai X. Z.; Li W. H.; Shi R.; Wei H. D.; Cao C.; Yu M.; Liu H.; Dong F. T.; Wang D. G.; Zhang X. M.; Qian X. H.; Zhu Q. Y.; He F. C. Proteomic analysis on structural proteins of Severe Acute Respiratory Syndrome coronavirus. Proteomics 2004, 4 (2), 492–504. 10.1002/pmic.200300676. 14760722 PMC7168022

[ref30] Diemer C.; Schneider M.; Seebach J.; Quaas J.; Frosner G.; Schatzl H. M.; Gilch S. Cell type-specific cleavage of nucleocapsid protein by effector caspases during SARS coronavirus infection. J. Mol. Biol. 2008, 376 (1), 23–34. 10.1016/j.jmb.2007.11.081. 18155731 PMC7094231

[ref31] Meyer B.; Chiaravalli J.; Gellenoncourt S.; Brownridge P.; Bryne D. P.; Daly L. A.; Walter M.; Agou F.; Chakrabarti L. A.; Craik C. S.; Eyers C. E.; Eyers P. A.; Gambin Y.; Sierecki E.; Verdin E.; Vignuzzi M.; Emmott E. Characterisation of protease activity during SARS-CoV-2 infection identifies novel viral cleavage sites and cellular targets for drug repurposing. BioRxiv 2021, 10.1101/2020.09.16.297945. PMC8455558 34548480

[ref32] Narayanan K.; Makino S. Cooperation of an RNA packaging signal and a viral envelope protein in coronavirus RNA packaging. J. Virol. 2001, 75 (19), 9059–9067. 10.1128/JVI.75.19.9059-9067.2001. 11533169 PMC114474

[ref33] Sarni S.; Biswas B.; Liu S. H.; Olson E. D.; Kitzrow J. P.; Rein A.; Wysocki V. H.; Musier-Forsyth K. HIV-1 Gag protein with or without p6 specifically dimerizes on the viral RNA packaging signal. J. Biol. Chem. 2020, 295 (42), 14391–14401. 10.1074/jbc.RA120.014835. 32817318 PMC7573273

[ref34] Masters P. S. Coronavirus genomic RNA packaging. Virology 2019, 537, 198–207. 10.1016/j.virol.2019.08.031. 31505321 PMC7112113

[ref35] Haller A. A.; Stewart S. R.; Semler B. L. Attenuation stem-loop lesions in the 5′ noncoding region of poliovirus RNA: Neuronal cell-specific translation defects. J. Virol. 1996, 70 (3), 1467–1474. 10.1128/jvi.70.3.1467-1474.1996. 8627664 PMC189967

[ref36] Dietrich L.; Ehrlich L. S.; LaGrassa T. J.; Ebbets-Reed D.; Carter C. Structural consequences of cyclophilin a binding on maturational refolding in human immunodeficiency virus type 1 capsid protein. J. Virol. 2001, 75 (10), 4721–4733. 10.1128/JVI.75.10.4721-4733.2001. 11312344 PMC114227

[ref37] He Y. X.; Zhou Y. S.; Wu H.; Kou Z. H.; Liu S. W.; Jiang S. B. Mapping of antigenic sites on the nucleocapsid protein of the severe acute respiratory syndrome coronavirus. J. Clin. Microbiol. 2004, 42 (11), 5309–5314. 10.1128/JCM.42.11.5309-5314.2004. 15528730 PMC525273

[ref38] Li S.; Lin L.; Wang H.; Yin J.; Ren Y.; Zhao Z.; Wen J.; Zhou C.; Zhang X.; Li X.; Wang J.; Zhou Z.; Liu J.; Shao J.; Lei T.; Fang J.; Xu N.; Liu S. The Epitope Study on the SARS-CoV Nucleocapsid Protein. *Genomics*. Genomics, Proteomics Bioinf. 2003, 1, 198–206. 10.1016/S1672-0229(03)01025-8. PMC5172353 15629032

[ref39] Shang B.; Wang X. Y.; Yuan J. W.; Vabret A.; Wu X. D.; Yang R. F.; Tian L.; Ji Y. Y.; Deubel V.; Sun B. Characterization and application of monoclonal antibodies against N protein of SARS-coronavirus. Biochem. Biophys. Res. Commun. 2005, 336 (1), 110–117. 10.1016/j.bbrc.2005.08.032. 16112641 PMC7092910

[ref40] Lee H. K.; Lee B. H.; Dutta N. K.; Seok S. H.; Baek M. W.; Lee H. Y.; Kim D. J.; Na Y. R.; Noh K. J.; Park S. H.; Kariwa H.; Nakauchi M.; Mai L. Q.; Heo S. J.; Park J. H. Detection of Antibodies Against SARS-Coronavirus Using Recombinant Truncated Nucleocapsid Proteins by ELISA. J. Microbiol Biotechn 2008, 18 (10), 1717–1721. 18955825

[ref41] Dai L. P.; Gao G. F. Viral targets for vaccines against COVID-19. Nat. Rev. Immunol. 2021, 21 (2), 73–82. 10.1038/s41577-020-00480-0. 33340022 PMC7747004

[ref42] Cong Y. Y.; Kriegenburg F.; de Haan C. A. M.; Reggiori F. Coronavirus nucleocapsid proteins assemble constitutively in high molecular oligomers. Sci. Rep. 2017, 7, 5740 10.1038/s41598-017-06062-w. 28720894 PMC5515880

[ref43] Softic L.; Brillet R.; Berry F.; Ahnou N.; Nevers Q.; Morin-Dewaele M.; Hamadat S.; Bruscella P.; Fourati S.; Pawlotsky J. M.; Ahmed-Belkacem A. Inhibition of SARS-CoV-2 Infection by the Cyclophilin Inhibitor Alisporivir (Debio 025). Antimicrob. Agents Chemother. 2020, 64 (7), e00876-20 10.1128/AAC.00876-20. 32376613 PMC7318051

[ref44] Rial D. V.; Ceccarelli E. A. Removal of DnaK contamination during fusion protein purifications. Protein Expression Purif. 2002, 25 (3), 503–507. 10.1016/S1046-5928(02)00024-4. 12182832

[ref45] DeHart C. J.; Fellers R. T.; Fornelli L.; Kelleher N. L.; Thomas P. M. Bioinformatics Analysis of Top-Down Mass Spectrometry Data with ProSight Lite. Methods Mol. Biol. 2017, 1558, 381–394. 10.1007/978-1-4939-6783-4_18. 28150248 PMC5373093

