# Supplementary material for: Multiple Roles of SARS-CoV-2 N Protein Facilitated by Proteoform-Specific Interactions with RNA, Host Proteins, and Convalescent Antibodies
Source: JACS Au. 2021 Jun 15;1(8):1147–57. doi: 10.1021/jacsau.1c00139 (PMC8231660; doi:10.1021/jacsau.1c00139)
Supplement: Supplementary file 1 — au1c00139_si_001.pdf [file au1c00139_si_001.pdf]

Supporting Information

Multiple roles of SARS-CoV-2 N protein facilitated by proteoform-specific interactions with RNA, host proteins, and convalescent antibodies

Corinne A. Lutomski[1], Tarick J. El-Baba[1], Jani R. Bolla[1], Carol V. Robinson[1]*

[1]*Physical and Theoretical Chemistry Laboratory, University of Oxford, South Parks Road, OX13QZ Oxford, UK*

*Corresponding author. Email: carol.robinson@chem.ox.ac.uk

This PDF file includes:

Extended Materials and Methods
Figures S1 to S16
Tables S1 to S7

Extended Materials and Methods

Size Exclusion Chromatography. Full-length N protein and N proteoforms were separated using a Superdex 10/300 increase GL column equilibrated in lysis buffer. Fractions corresponding to N_{FL} and N proteoforms were pooled and concentrated to ~10 µM before MS analysis.

Liquid Chromatography and Bottom-up Mass Spectrometry. Full-length N was separated from the lower molecular weight proteoforms on a 0.8 mm 4-12% bis-tris SDS-PAGE gel (Invitrogen) and stained with Coomassie Blue. Bands were excised from the gel, minced, and digested with sequencing grade trypsin (Promega, Madison, WI, U.S.A) at 37 °C overnight, extracted with 80% acetonitrile (0.1% formic acid), and dried on a vacuum concentrator. The extracted peptides were resolubilized in buffer A (H_2O, 0.1% FA) and loaded onto a reverse phase trap column (Acclaim PepMap 100, 75µm x 2 cm, nano viper, C18, 3 µm, 100 Å, ThermoFisher, Waltham, MA, U.S.A.) using an Ultimate 3000 for 50 µL at a flow rate of 10 µL min^{-1}. The trapped peptides were then separated using a 15 cm reverse phase analytical column (350 µm x 75 µm) packed in-house (3 µm C18 particles) using a 60 min linear gradient from 5% to 40% buffer B (80% acetonitrile, 20% water, 0.1% formic acid) at a flow rate of 300 nL min^{-1}. The separated peptides were then electrosprayed in the positive ion mode into an Orbitrap Eclipse Tribrid mass spectrometer (ThermoFisher, San Jose, CA, USA) operated in data-dependent acquisition mode (3 s cycle time). Precursor and product mass analysis occurred in the Orbitrap analyzer (120,000 and 60,000 resolving power at *m/z* 200, respectively). High intensity (threshold: 1.0 x 10^4) precursors with charge state between z = 2 and z = 7 were isolated with the quadrupole (0.5 *m/z* offset, 0.7 *m/z* isolation window) and fragmented using higher energy collision induced dissociation (HCD collision energy = 30%). Additional MS/MS scans for precursors within 10 ppm were dynamically excluded for 30 s following the initial selection. MS/MS scans were collected using an automated gain control setting of 1.0 x 10^4 or a maximum fill time of 100 ms. LC-MS data were searched against both the *E. coli* proteome manually annotated with the SARS-CoV-2 nucleocapsid protein sequence using MaxQuant v1.6.17.0.

Expression in Mammalian Cells. The codon-optimized cDNA for full-length N protein with an N-terminal FLAG tag followed by a tobacco etch virus cleavage sequence was cloned into pcDNA5/TO (Gibco, Thermo Fisher Scientific) using restriction cloning. Codon optimized cDNA for the membrane (M) protein, and envelope (E) proteins of SARS-CoV-2 containing C-terminal FLAG tags were cloned into pcDNA5/TO using restriction cloning. Human embryonic kidney (HEK) 293T cells (American Type Culture Collection, ATCC CRL-3216) were seeded in two 6-well plates 24 hours prior to transfection. In one plate, the pcDNA5/TO plasmid containing the N protein gene was transfected into HEK293T cells using Lipofectamine 2000 in a ratio of 1:1 DNA:Lipofectamine and a final concentration of 2 µg DNA per well. In the second well plate, plasmids containing N, M, and E proteins were co-transfected using Lipofectamine 2000 in a ratio of 1:0.5:0.5:2 N:M:E:DNA with a final concentration of 2 µg DNA per well. Cells were maintained at 37 °C, 5% CO_2, in Opti-MEM Reduced Serum Media (Gibco) for 24 hours. After 24 hours, cell media was changed to DMEM:F12 (Gibco), supplemented with 10% FBS (Life Technologies) and non-essential amino acids (Gibco). Cells were harvested 48 hours after transfection and used for Western Blot analysis.

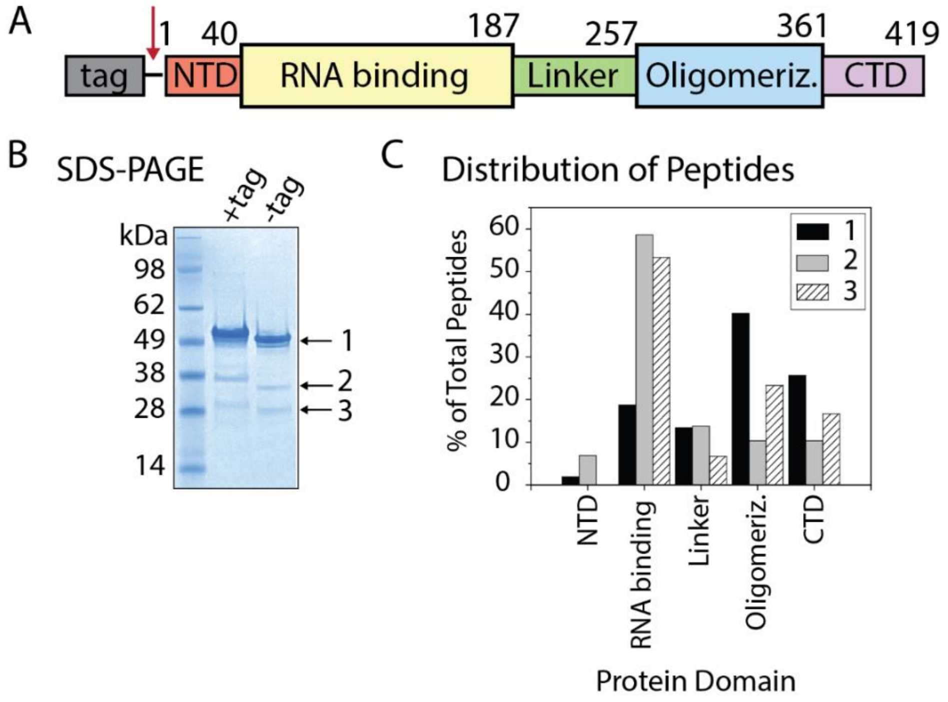

Figure S1. Scheme depicting the full-length construct for expression in *E. coli*. The construct contains an N-terminal hexahistidine tag followed by a TEV cleavage motif. The tag was cleaved at the site indicated by the red arrow. (B) SDS-PAGE of the N protein construct in (A) before and after tag cleavage. Before removal of the purification tag, three distinct protein bands were detected at ~49, ~38 and ~28 kDa. Following removal of the tag, all three protein bands migrated by ~3 kDa, or the mass of the tag. (C) The protein bands were subjected to in-gel digestion with trypsin followed by LC-MS based proteomics. We confirmed that each band corresponded to N protein by in-gel trypsin digestion followed by LC-MS-based bottom-up proteomics. All three bands contained peptides from the N protein, resulting in 78.1%, 49.9%, and 43.2% sequence coverage for bands 1, 2, and 3, respectively. We observe an unbiased distribution of peptides across all five domains for band 1, consistent with the expectation that tryptic peptides would be reasonably distributed across the full-length protein. Over 50% of the total peptides detected in bands 2 and 3 were localized to the RNA binding domain, suggesting that the proteins are predominantly N-terminal derivatives. In all three bands, peptides located in the 58-residue C-terminal domain were detected, indicating the purified protein is made up of a diverse mixture of N proteoforms[1] and not biased to N-terminal species due to the location of the purification tag. The histogram displays the percentage of peptides detected, relative to the total peptide count, across the five protein domains: N-terminal domain (NTD), RNA binding, Linker, Oligomerization, and C-terminal Domain (CTD).

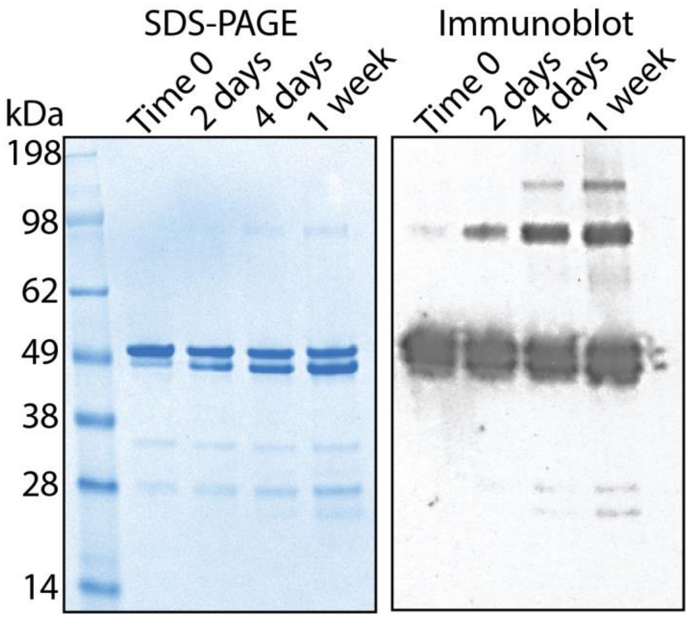

Figure S2. N proteoforms are consistently generated over time. (Left) SDS-PAGE and (Right) Western Blot of isolated N protein analyzed at 2 days, 4 days, and 1 week after incubation at room temperature. A monoclonal antibody raised against the full-length N protein was used to blot against the proteins in the panel on the right. The SDS-PAGE and Western blot reveal distinct bands that change in intensity (brightness) over the course of one week. The temporal decrease in brightness for the band corresponding to the full-length N protein, concomitant with the increase in intensity for the truncated proteoforms, suggest that the proteoforms are continuously generated.

Table S1. Statistical analysis for HCD fragmentation products matched to candidate sequences.

Deconvoluted mass of charge state distribution: 22,611 ± 1

Tentative Assignment: $N_{1\text{-}209}$

Candidate Sequence	Sequence Mass (Da)	Precursor Charge State	Fragments matched	P-score	Coverage (%)
N_{FL}	45,769.83	8+	0	1	0
		9+	10	1.40e-9	2
		10+	0	1	0
$N_{1\text{-}208}$	22,456.52	8+	0	1	0
		9+	10	1.40e-9	5
		10+	0	1	0
**$N_{1\text{-}209}$**	**22,612.71**	8+	0	1	0
		**9+**	**11**	**7.60e-11**	**5**
		10+	1	0.07	0
$N_{1\text{-}210}$	22,743.9	8+	0	1	0
		9+	9	2.20e-8	4
		10+	0	1	0
$N_{215\text{-}419}$	22,687.6	8+	0	1	0
		9+	1	4.60e-1	0
		10+	0	1	0
$N_{216\text{-}419}$	22,572.51	8+	0	1	0
		9+	1	4.60e-1	0
		10+	0	1	0

N G S M S D N G P Q N Q R N A P R I I T F G G P S D S 25
26 T G S N Q N G E R S G A R S K Q R R P Q G L P N N 50
51 T A S W F T A L T Q H G K E D L K F P R G Q G V P 75
76 I N T N S S P D D Q I G Y Y R R A T R R I R G G D 100
101 G K M K D L S P R W Y F Y Y L G T G P E A G L P Y 125
126 G A N K D G I I W V A T E G A L N T P K D H I G T 150
151 R N P A N N A A I V L Q L P Q G T T L P K G F Y A 175
176 E G S R G G S Q A S S R S S S R S R N S S R N S T 200
201 P G S S R G T S P A R C

Table S2. Statistical analysis for HCD fragmentation products matched to candidate sequences.

Deconvoluted mass of charge state distribution: 23,540 ± 0.3

Tentative Assignment: N_{1-220}

Candidate Sequence	Sequence Mass (Da)	Precursor Charge State	Fragments matched	P-score	Sequence Coverage (%)
N_{FL}	45,769.83	9+	5	3.00e-3	1
		10+	0	1	0
N_{1-219}	23,470.65	9+	5	3.00e-3	2
		10+	0	1	0
**N_{1-220}**	23,541.73	**9+**	**9**	**7.60e-7**	**4**
		10+	1	0.07	0
N_{1-221}	23,654.89	9+	5	3.00e-3	2
		10+	0	1	0
$N_{206-419}$	23,499.52	9+	1	0.61	0
		10+	0	1	0
$N_{205-419}$	23,586.59	9+	1	0.61	0
		10+	0	1	0

N G S M S D N G P Q N Q R N A P R I T F G G P S D S 25
26 T G S N Q N G E R S G A R S K Q R R P Q G L P N N 50
51 T A S W F T A L T Q H G K E D L K F P R G Q G V P 75
76 I N T N S S P D D Q I G Y Y R R A T R R I R G G D 100
101 G K M K D L S P R W Y F Y Y L G T G P E A G L P Y 125
126 G A N K D G I I W V A T E G A L N T P K D H I G T 150
151 R N P A N N A A I V L Q L P Q G T T L P K G F Y A 175
176 E G S R G G S Q A S S R S S S R S R N S S R N S T 200
201 P G S S R G T S P A R M A G N G G D A A L A C

Table S3. Statistical analysis for HCD fragmentation products matched to candidate sequences.

Deconvoluted mass of charge state distribution: 29,402 ± 0.6*
Tentative Assignment: N_{1-273}

Candidate Sequence	Sequence Mass (Da)	Precursor Charge State	Fragments matched	P-score	Sequence Coverage (%)
N_{FL}	45,769.83				
		10+	0	1	0
		11+	3	2.0e-2	0
N_{1-272}	29,311.35				
		10+	0	1	0
		11+	3	2.0e-2	0
**N_{1-273}**	29,382.43				
		10+	0	1	0
		**11+**	6	2.90e-5	2
N_{1-274}	29,529.60				
		10+	0	1	0
		11+	3	2.0e-2	0
$N_{148-419}$	29,433.89				
		10+	1	0.4	0
		11+	0	1	0
$N_{149-419}$	29,277.70				
		10+	2	0.095	1
		11+	0	1	0

N G S M S D N G P Q N Q R N A P R I T F G G P S D S 25
26 T G S N Q N G E R S G A R S K Q R R P Q G L P N N 50
51 T A S W F T A L T Q H G K E D L K F P R G Q G V P 75
76 I N T N S S P D D Q I G Y Y R R A T R R I R G G D 100
101 G K M K D L S P R W Y F Y Y L G T G P E A G L P Y 125
126 G A N K D G I I W V A T E G A L N T P K D H I G T 150
151 R N P A N N A A I V L Q L P Q G T T L P K G F Y A 175
176 E G S R G G S Q A S S R S S S R S R N S S R N S T 200
201 P G S S R G T S P A R M A G N G G D A A L A L L L 225
226 L D R L N Q L E S K M S G K G Q Q Q Q G Q T V T K 250
251 K S A A E A S K K P R Q K R T A T K A Y N V T Q A C

*The discrepancy in the deconvoluted mass and sequence mass is expected to arise from methionine oxidation. Proteomics analysis of tryptic peptides of N proteoforms has detected five oxidized methionines at positions 3, 103, and 212, however sites 103 and 212 reside in the interior of the protein with no available sequence coverage, we were unable to localize the exact site of modification.

Table S4. Statistical analysis for HCD fragmentation products matched to candidate sequences.

Deconvoluted mass of charge state distribution: 28,735 ± 2*

Tentative Assignment: $N_{156\text{-}419}$

Candidate Sequence	Sequence Mass (Da)	Precursor Charge State	Fragments matched	P-score	Sequence Coverage (%)
N_{FL}	45,769.83				
		10+	1	0.54	0
		11+	0	1	0
$N_{1\text{-}266}$	28,634.62				
		10+	0	1	0
		11+	0	1	0
$N_{1\text{-}267}$	28,705.70				
		10+	0	1	0
		11+	0	1	0
$N_{1\text{-}268}$	28,868.88				
		10+	0	1	0
		11+	0	1	0
$N_{154\text{-}419}$	28,881.30				
		10+	1	0.54	0
		11+	0	1	0
$N_{155\text{-}419}$	28,767.20				
		10+	1	0.54	0
		11+	0	1	0
**$N_{156\text{-}419}$**	**28,696.12**				
		**10+**	**2**	**0.18**	**1**
		11+	0	1	0

N A I V L Q L P Q G T T L P K G F Y A E G S R G G S 25
26 Q A S S R S S S R S R N S S R N S T P G S S R G T 50
51 S P A R M A G N G G D A A L A L L L L D R L N Q L 75
76 E S K M S G K G Q Q Q Q G Q T V T K K S A A E A S 100
101 K K P R Q K R T A T K A Y N V T Q A F G R R G P E 125
126 Q T Q G N F G D Q E L I R Q G T D Y K H W P Q I A 150
151 Q F A P S A S A F F G M S R I G M E V T P S G T W 175
176 L T Y T G A I K L D D K D P N F K D Q V I L L N K 200
201 H I D A Y K T F P P T E P K K D K K K K A D E T Q 225
226 A L P Q R Q K K Q Q T V T L L P A A D L D D F S K 250
251 Q L Q Q S M S S A D S T Q A C

*The discrepancy in the deconvoluted mass and sequence mass is expected to arise from methionine oxidation. Proteomics analysis of tryptic peptides of N proteoforms has detected five oxidized methionines at positions 212, 319, 324, and 419, however sites 212, 319, and 324 reside in the interior of the protein with no available sequence coverage, we were unable to localize the exact site of modification.

Table S5. Statistical analysis for HCD fragmentation products matched to candidate sequences.

Deconvoluted mass of charge state distribution: 42,922 ± 1
Tentative Assignment: $N_{1\text{-}392}$

Candidate Sequence	Sequence Mass (Da)	Precursor Charge State	Fragments matched	P-score	Sequence Coverage (%)
N_{FL}	45,769.83	13+	0	1	0
		12+	1	1.8e-1	0
$N_{1\text{-}391}$	42,718.50	13+	0	1	0
		12+	1	1.8e-1	0
$N_{1\text{-}392}$	42,918.74	13+	12	6.1e-14	4
		12+	10	2.1e-11	3
$N_{1\text{-}393}$	43,019.84	13+	1	0.19	0
		12+	2	1.8e-2	1
$N_{24\text{-}419}$	43,096.02	13+	0	1	0
		12+	0	1	0

N G S M S D N G P Q N Q R N A P R I T F G G P S D S 25
26 T G S N Q N G E R S G A R S K Q R R P Q G L P N N 50
51 T A S W F T A L T Q H G K E D L K F P R G Q G V P 75
76 I N T N S S P D D Q I G Y Y R R A T R R I R G G D 100
101 G K M K D L S P R W Y F Y Y L G T G P E A G L P Y 125
126 G A N K D G I I W V A T E G A L N T P K D H I G T 150
151 R N P A N N A A I V L Q L P Q G T T L P K G F Y A 175
176 E G S R G G S Q A S S R S S S R S R N S S R N S T 200
201 P G S S R G T S P A R M A G N G G D A A L A L L L 225
226 L D R L N Q L E S K M S G K G Q Q Q Q G Q T V T K 250
251 K S A A E A S K K P R Q K R T A T K A Y N V T Q A C

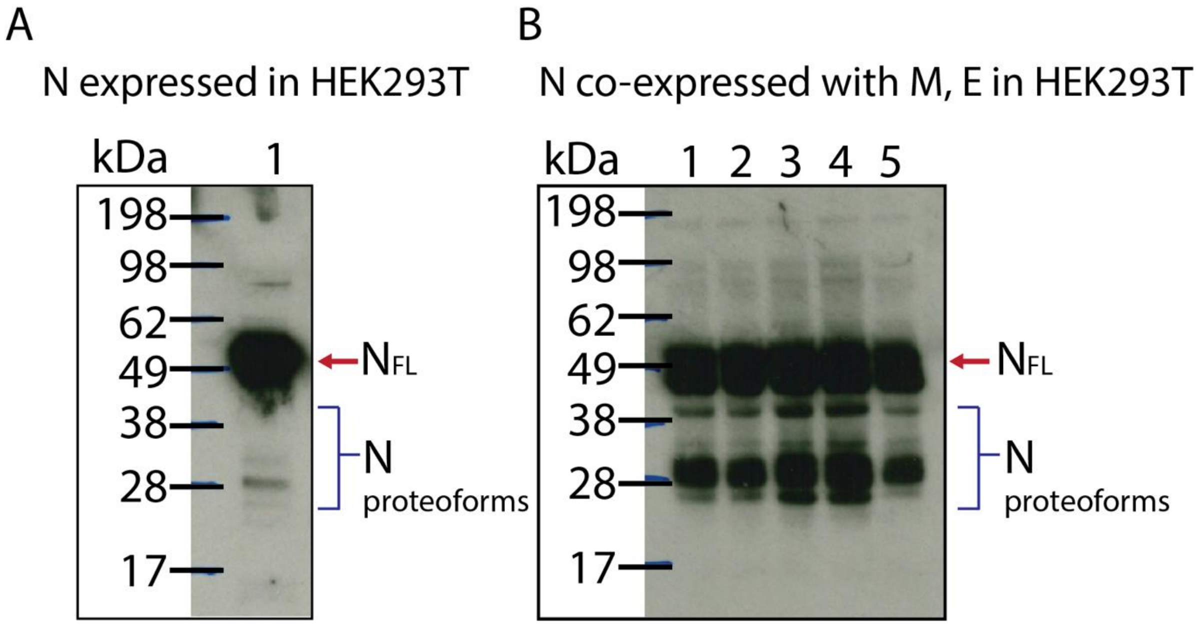

Figure S3. N proteoforms are enhanced in whole cells lysates following co-expression with membrane (M) and envelope (E) proteins of SARS-CoV-2 in HEK 293T cells. Western blot from HEK293T cells (A) stably expressing full-length N protein and (B) co-expressing N, M, and E proteins. In both Western Blots, the primary antibody used was a recombinant monoclonal antibody raised against the highly homologous SARS-CoV nucleocapsid protein) with cross-reactivity to the SARS-CoV-2 N protein. Lanes numbered 1 through 5 in (B) represent replicates from five wells transfected with M, E, and N proteins.

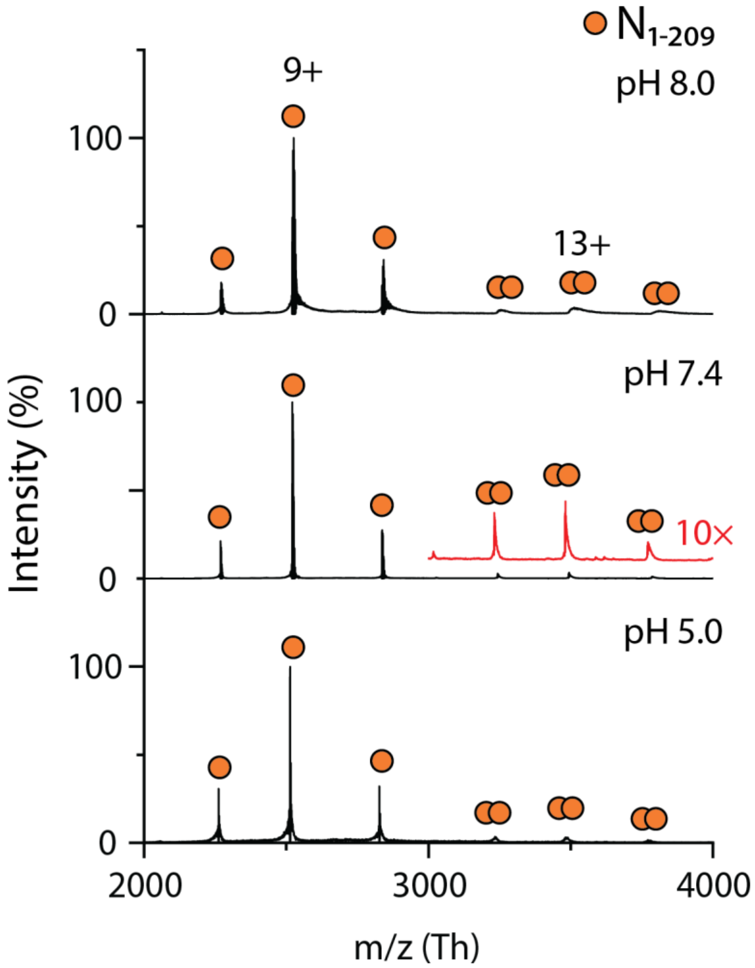

Figure S4. The oligomeric state of $N_{1\text{-}209}$ is not influenced by pH. Mass spectra of $N_{1\text{-}209}$ measured at pH 8.0, 7.4 and 5.0 (top to bottom). Two charge state distributions are observed that correspond to monomers and dimers of $N_{1\text{-}209}$, centered at 9+ and 13+, respectively. The mass spectrum recorded at pH 7.4 was magnified 10× at *m/z* 3000 and offset for clarity (red trace) to highlight the low abundance distribution of $N_{1\text{-}209}$ dimer. The deconvoluted masses of each distribution can be found in Table S6.

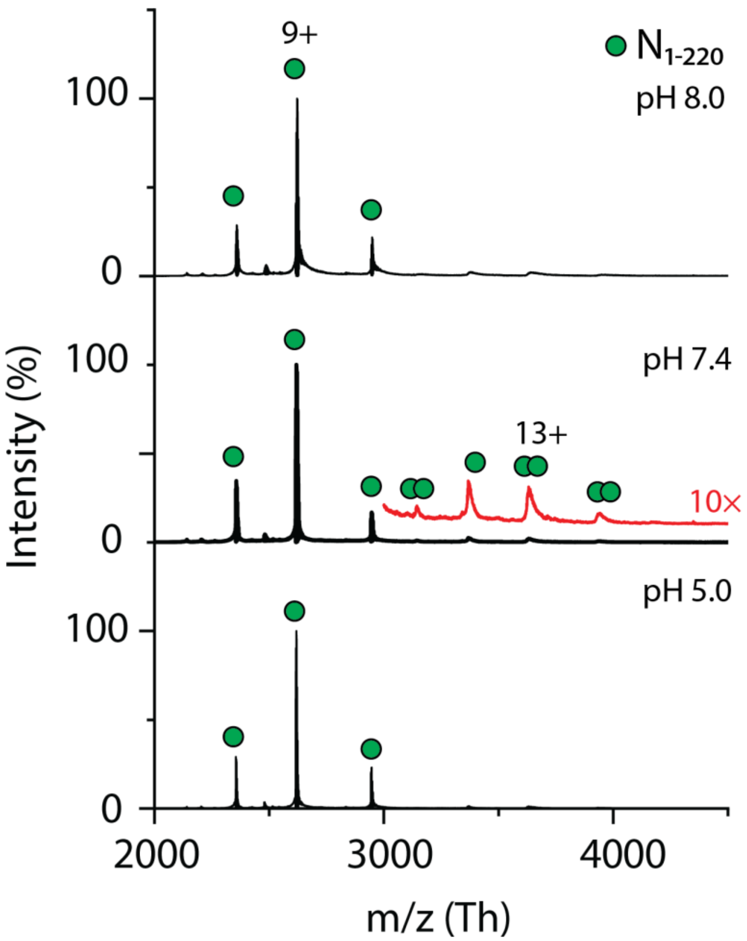

Figure S5. The oligomeric state of N_{1-220} is not influenced by pH. Mass spectra of N_{1-220} measured at pH 8.0, 7.4 and 5.0 (top to bottom). Two charge state distributions are observed that correspond to monomers and dimers of N_{1-220}, centered at 9+ and 13+, respectively. The mass spectrum recorded at pH 7.4 was magnified 10× at *m/z* 3000 and offset for clarity (red trace) to highlight the low abundance distribution of N_{1-220} dimer. The deconvoluted masses of each distribution can be found in Table S6.

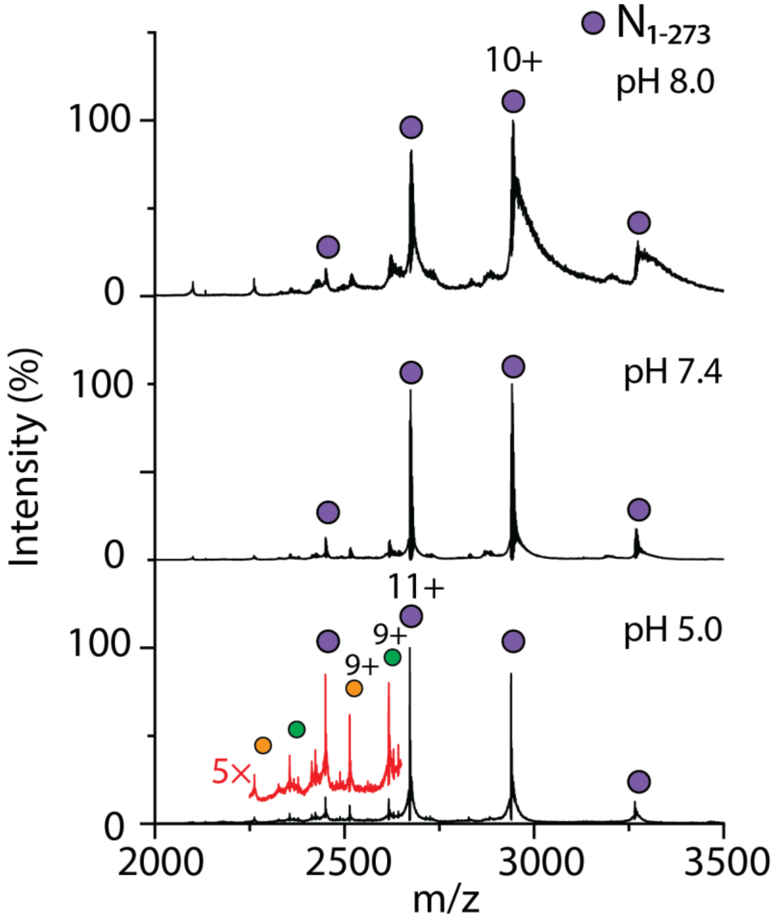

Figure S6. The oligomeric state of $N_{1\text{-}273}$ is not influenced by pH. Mass spectra of $N_{1\text{-}273}$ measured at pH 8.0, 7.4 and 5.0 (top to bottom). One charge state distribution is observed that correspond to monomeric $N_{1\text{-}273}$, centered at 10+ at pH 8.0 and 7.4, and 11+ at pH 5.0. The mass spectrum recorded at pH 5.0 was magnified 5× at *m/z* 2250-2650 and offset for clarity (red trace) to highlight the low abundance charge state distributions corresponding to $N_{1\text{-}209}$ and $N_{1\text{-}220}$ proteoforms resulting from the continued proteolytic cleavage of $N_{1\text{-}273}$. The deconvoluted masses of each distribution can be found in Table S6.

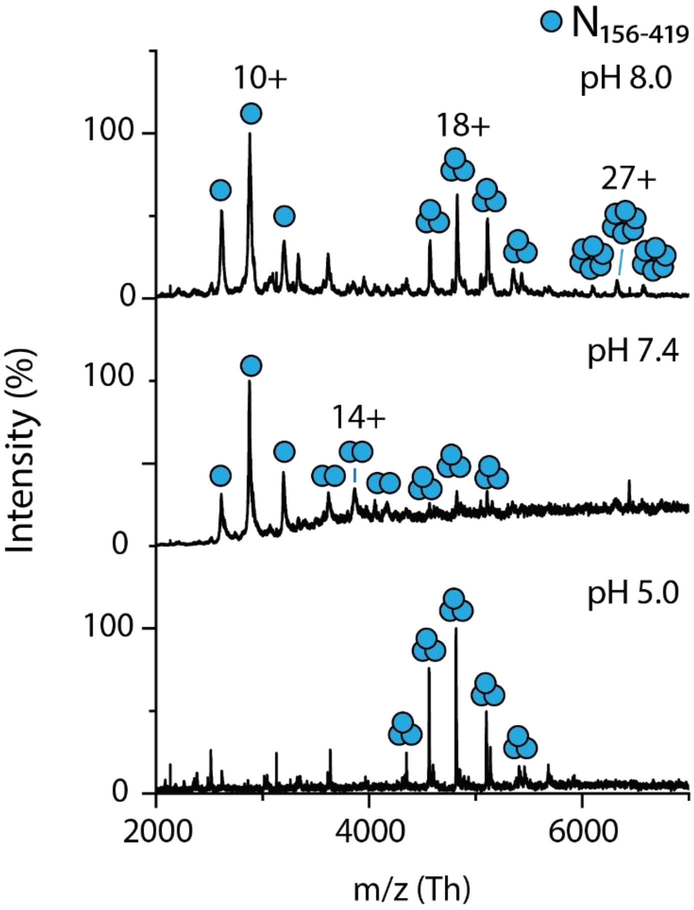

Figure S7. pH influences the oligomeric state of $N_{156-419}$. Mass spectra of $N_{156-419}$ measured at pH 8.0, 7.4, and 5.0 (top to bottom). $N_{156-419}$ appears to exhibit a pH-dependence on oligomeric state. At pH 8.0, the mass spectrum reveals three charge state distributions corresponding to monomers, trimers, and hexamers centered charge states 10+, 18+ and 27+, respectively. At pH 7.4, three charge state distributions corresponding to monomers, dimers and trimers are observed. At pH 5.0, only one charge state distribution is observed, indicating that $N_{156-419}$ is exclusively trimeric at pH 5.0. The deconvoluted masses of each distribution can be found in Table S6.

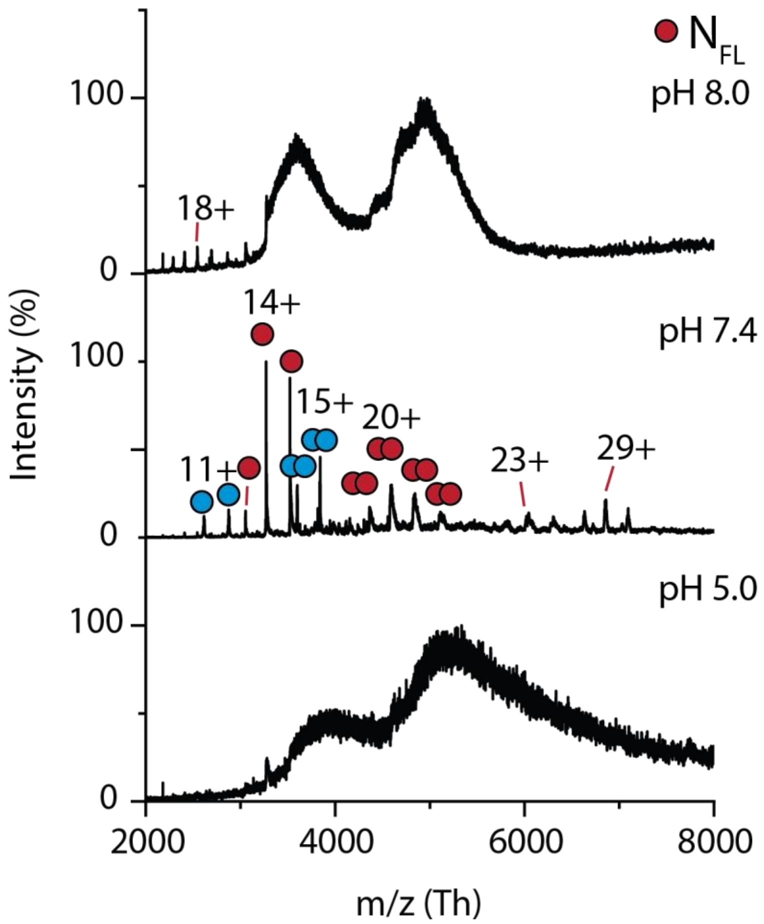

Figure S8. Effect of pH on the oligomeric state of N_{FL}. Mass spectra of N_{FL} measured at pH 8.0, 7.4 and 5.0 (top to bottom). At pH 7.4, several charge state distributions are observed that correspond to monomers and dimers of NFL (red circles with charges 14+ and 20+, respectively), monomers and dimers of N156-419 (blue circles with charges of 11+ and 15+, respectively) and two remaining charge state distributions centered at 23+ and 29+ that may correspond to hetero-oligomers of N_{FL} and N proteoforms. The deconvoluted masses of each distribution can be found in Table S6. The mass spectra at pH 5.0 and 8.0 reveal broadened and featureless peaks suggesting that it is likely aggregated. A low abundance series of highly charged peaks centered at 18+ at pH 8.0 indicates some protein unfolding.

Table S6. Deconvoluted and expected masses of assigned charge state distributions in Figures S3-S6

Protein	Deconvoluted Mass* ± s.d. (Da)	Sequence Mass (Da)
$N_{1\text{-}209}$	22,611 ± 1	22,612.71
$N_{1\text{-}209}$ dimer	45,225 ± 2	45,225.42
$N_{1\text{-}220}$	23,540 ± 0.3	23,541.73
$N_{1\text{-}220}$ dimer	47,106 ± 1	47,083.46
$N_{1\text{-}273}$	29,402 ± 0.6	29,382.43
$N_{156\text{-}419}$	28,735 ± 2	28,696.12
$N_{156\text{-}419}$ dimer	57,534 ± 1	57,392.24
$N_{156\text{-}419}$ trimer	86,700 ± 13	86,088.36
$N_{156\text{-}419}$ hexamer	170,813 ± 34	172,176.72
N_{FL}	45,769 ± 2	45,769.83
N_{FL} dimer	91,537 ± 2	91,539.66
Distribution centered at +23	144,958 ± 28	--
Distribution centered at +29	205,634 ± 23	--

* Determined using three adjacent charge states

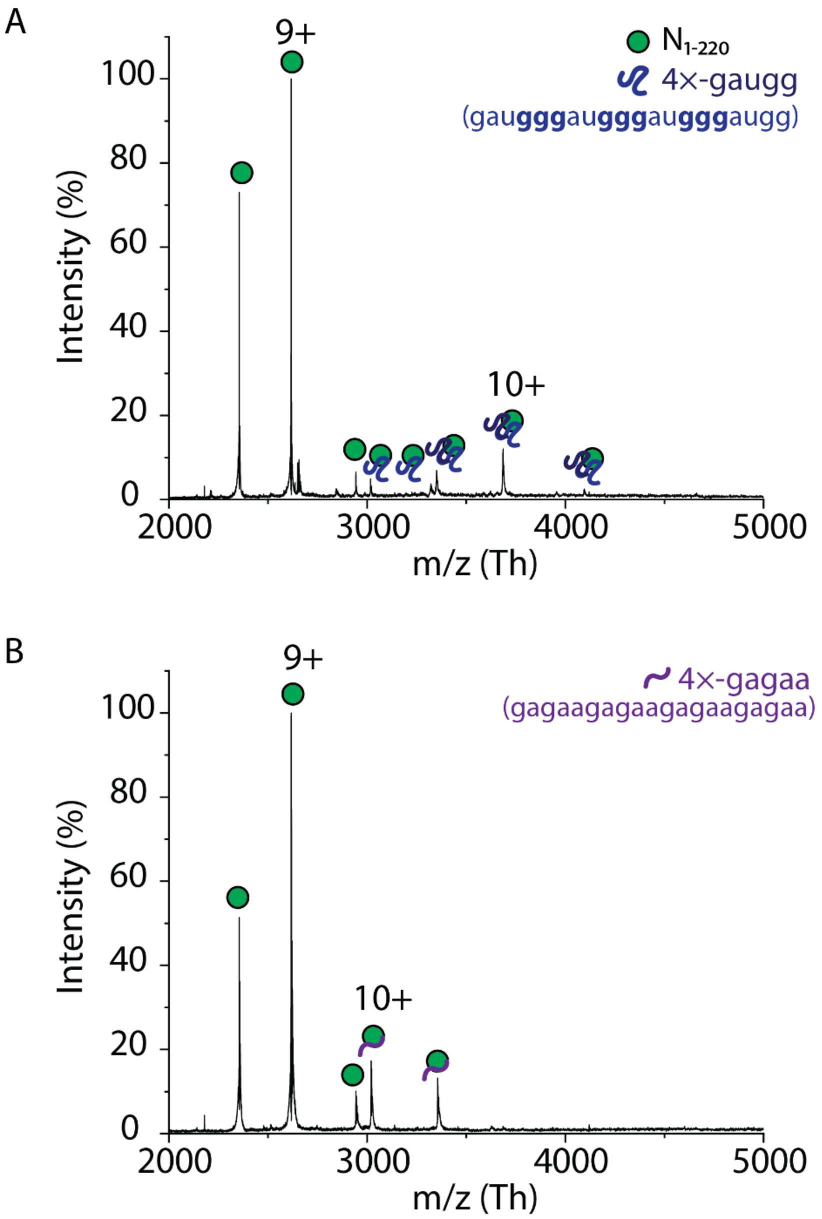

Figure S9. The RNA sequence influences binding stoichiometry to N_{1-220}. (A) Mass spectrum of N_{1-209} after incubation with 4×-GAUGG RNA oligonucleotides in a molar ratio of 1:4. The most abundant charge state distribution corresponds to monomeric N_{1-220} centered at the 9+ charge state. Two additional charge state distributions are observed above *m/z* 3000 that correspond to one and two RNA oligonucleotides bound to N_{1-220}. (B) Mass spectrum of N_{1-220} after incubation with 4×-GAGAA RNA oligonucleotides in a molar ratio of 1:4. The most abundant charge state distribution corresponds to monomeric N_{1-220} centered at the 9+ charge state. An additional, low abundant charge state series is observed between *m/z* 3000 and 3500 that corresponds to one RNA oligonucleotide bound to N_{1-220}.

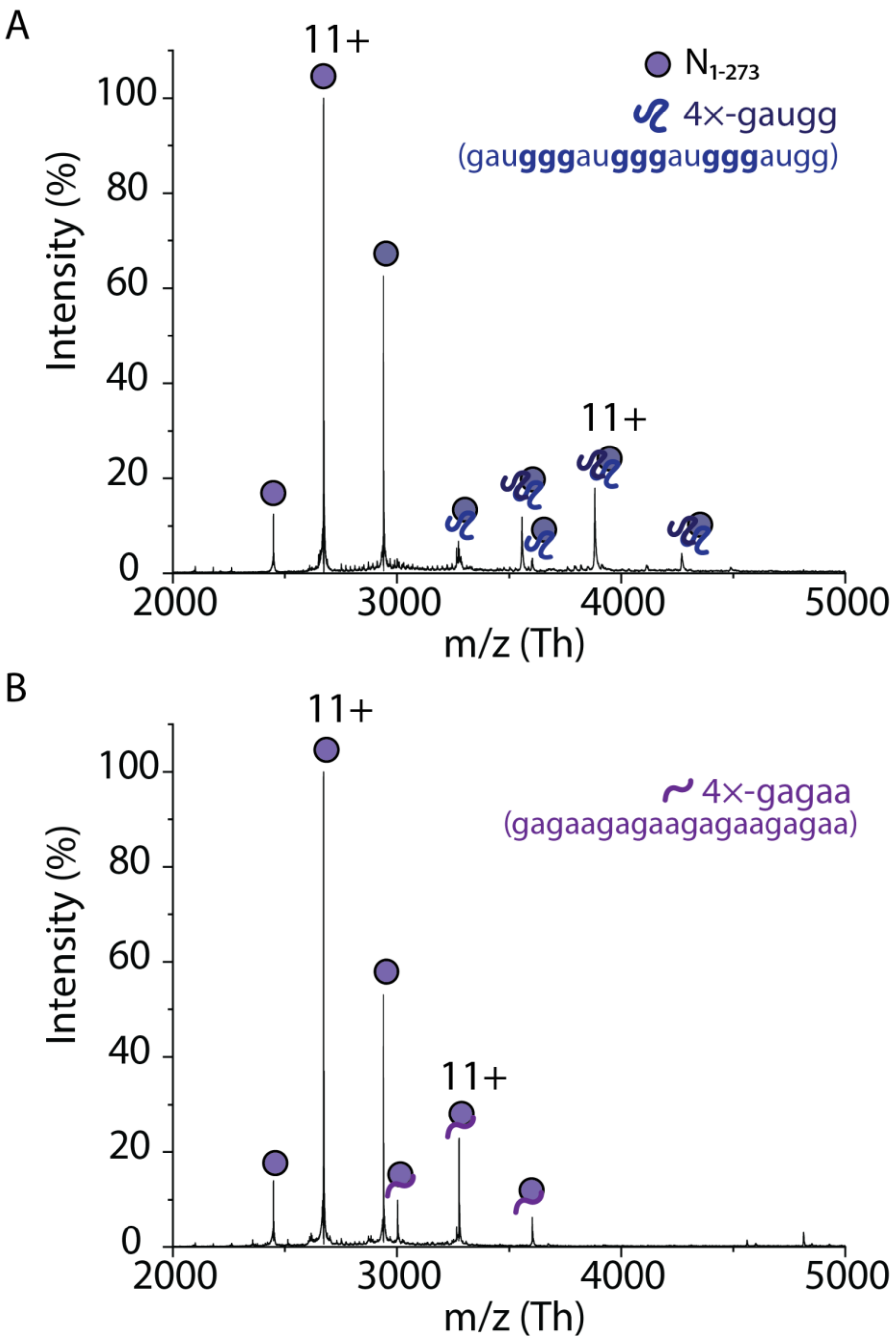

Figure S10. The RNA sequence influences binding stoichiometry to N_{1-273}. (A) Mass spectrum of N_{1-273} after incubation with 4×-GAUGG RNA oligonucleotides in a molar ratio of 1:4. The most abundant charge state distribution corresponds to monomeric N_{1-273} centered at the 11+ charge state. Two additional charge state distributions are observed above *m/z* 3000 that correspond to one and two RNA oligonucleotides bound to N_{1-273}. (B) Mass spectrum of N_{1-273} after incubation with 4×-GAGAA RNA oligonucleotides in a molar ratio of 1:4. The most abundant charge state distribution corresponds to monomeric N_{1-273} centered at the 11+ charge state. An additional, low abundant charge state series is observed between *m/z* 3000 and 4000 and centered at a charge state of 11+ that corresponds to one RNA oligonucleotide bound to N_{1-273}.

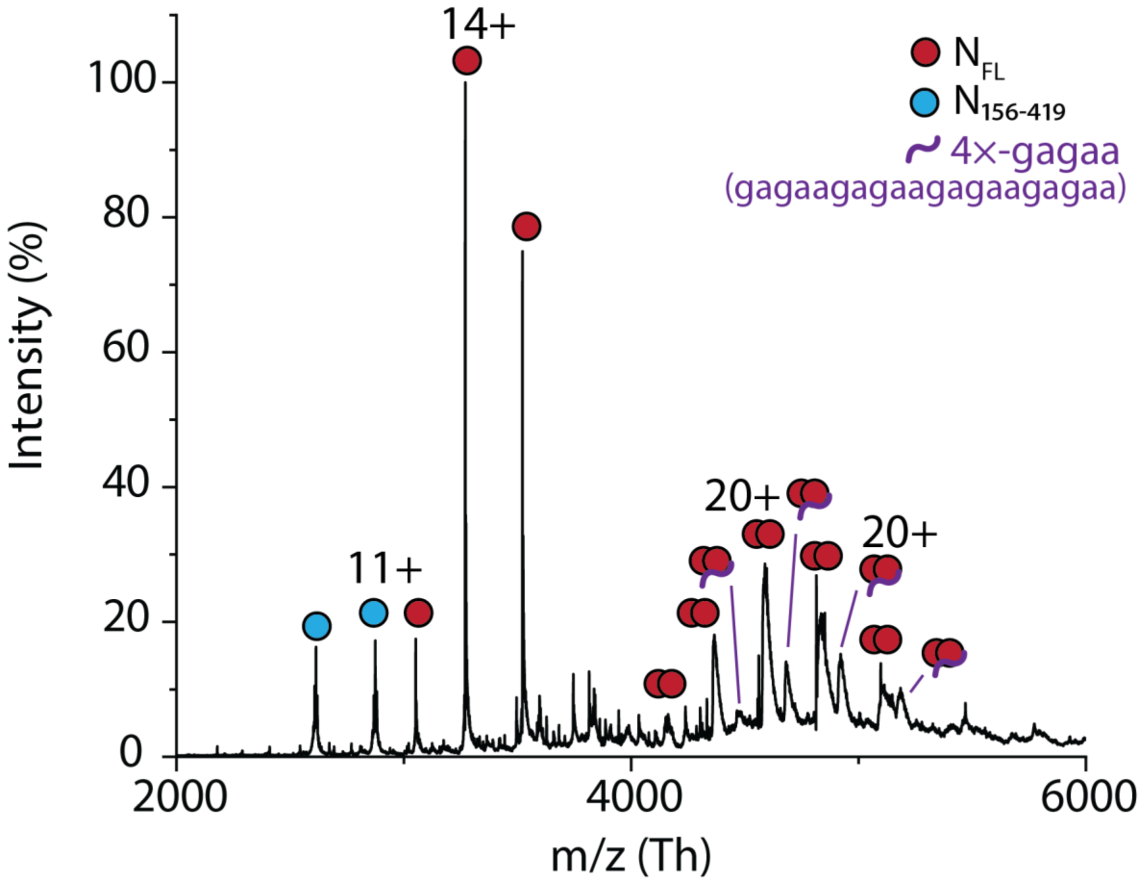

Figure S11. Mass spectrum of N_{FL} after incubation with 4×-GAGAA RNA oligonucleotides in a molar ratio of 1:4. Monomers and dimers of N_{FL} (red circles) and $N_{156-419}$ (blue circles) are observed. An additional peak series between 4300 and 5500 *m/z* corresponds to one 4×-gagaa RNA oligonucleotide bound to N_{FL} dimer.

Table S7. Deconvoluted and sequence masses of N protein-RNA complexes.

**Protein/Complex**	**Deconvoluted Mass ± s.d. (Da)**	**Sequence Mass (Da)**
4×-GAUGG RNA	--	6,622.00
4×-GAGAA RNA	--	6,650.20
N_{1-220} + one 4×-GAUGG RNA	30,162 ± 1.0	30,163.73
N_{1-220} + two 4×-GAUGG RNA	36,847 ± 13	36,785.73
N_{1-220} + one 4×-GAGAA RNA	30,191 ± 0.2	30,191.93
N_{1-273} + one 4×-GAUGG RNA	36,022 ± 27	36,004.43
N_{1-273} + two 4×-GAUGG RNA	42,696 ± 1.0	42,626.43
N_{1-273} + one 4×-GAGAA RNA	36,031 ± 0.5	36,032.63
N_{FL} dimer + 4×-GAGAA RNA	98,255 ± 46	98,189.86

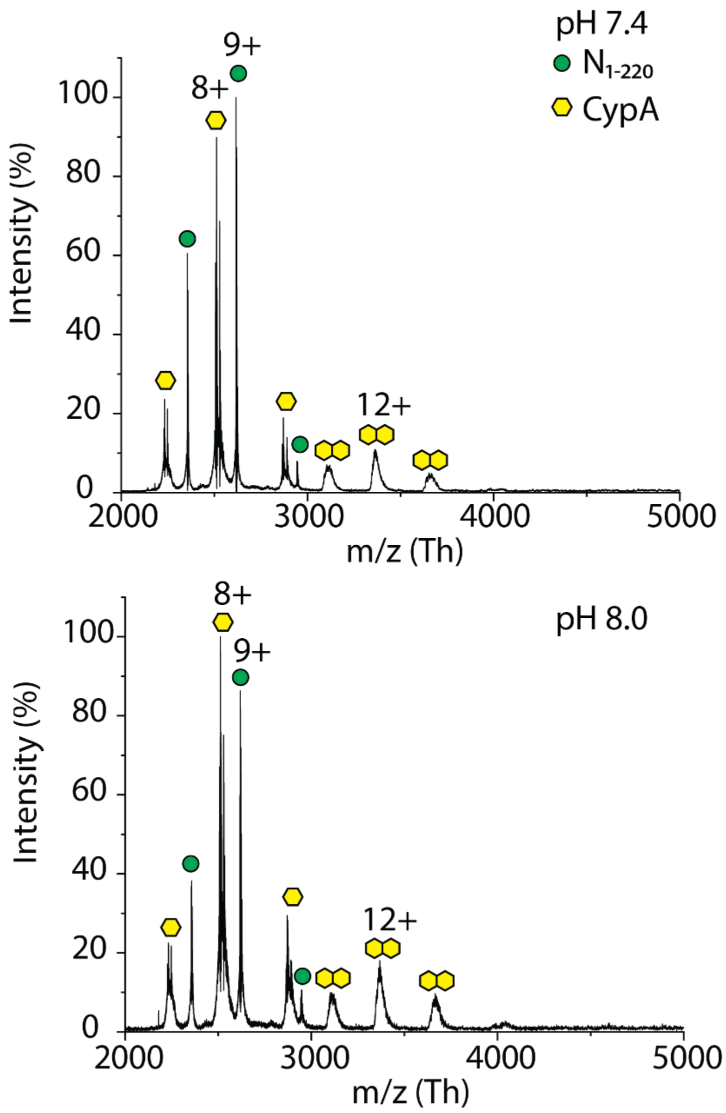

Figure S12. $N_{1\text{-}220}$ does not interact with cyclophilin A. Mass spectra show $N_{1\text{-}220}$ after incubation with cyclophilin A (CypA) in a 1:1 molar ratio at pH 7.4 (top) and pH 8.0 (bottom). In both spectra, three charge state distributions are observed: (i) a high abundant charge state distribution centered at 9+ that corresponds to $N_{1\text{-}220}$ monomers, (ii) a second highly abundant charge state distribution centered at 8+ that corresponds to CypA, and (iii) a low abundant charge state distribution at *m/z* >3000 and centered at 12+ that corresponds to CypA dimers. No interaction is observed between $N_{1\text{-}220}$ and CypA.

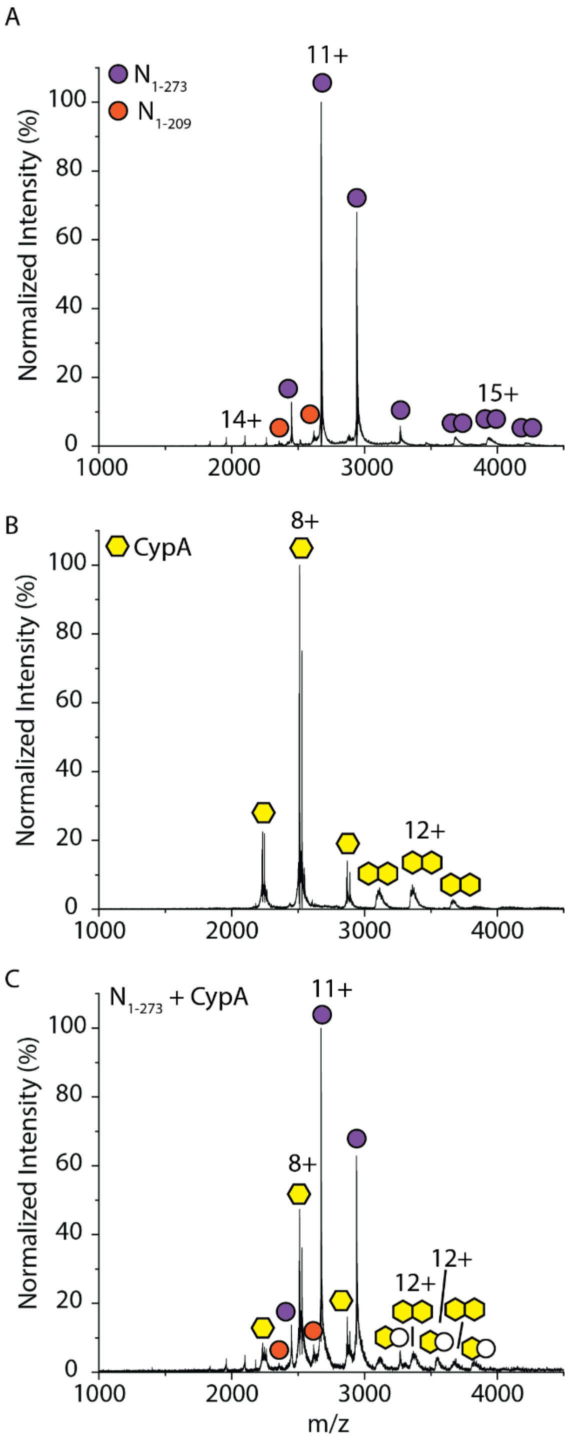

Figure S13. $N_{1\text{-}273}$ does not interact with cyclophilin A. (A) Mass spectrum of $N_{1\text{-}273}$ showing the low abundance charge state distributions corresponding to $N_{1\text{-}209}$ resulting from the continued proteolytic cleavage. (B) Mass spectrum of cypA showing a mixture of monomers and dimers and (C) Mass spectrum of $N_{1\text{-}273}$ after incubation with cyclophilin A (CypA) in a 1:1 molar ratio. The charge states >3000 m/z are broad and therefore the assignment cannot be unambiguously assigned to heterodimers of $N_{1\text{-}273}$:CypA due to the presence of $N_{1\text{-}209}$, a known interactor of CypA.

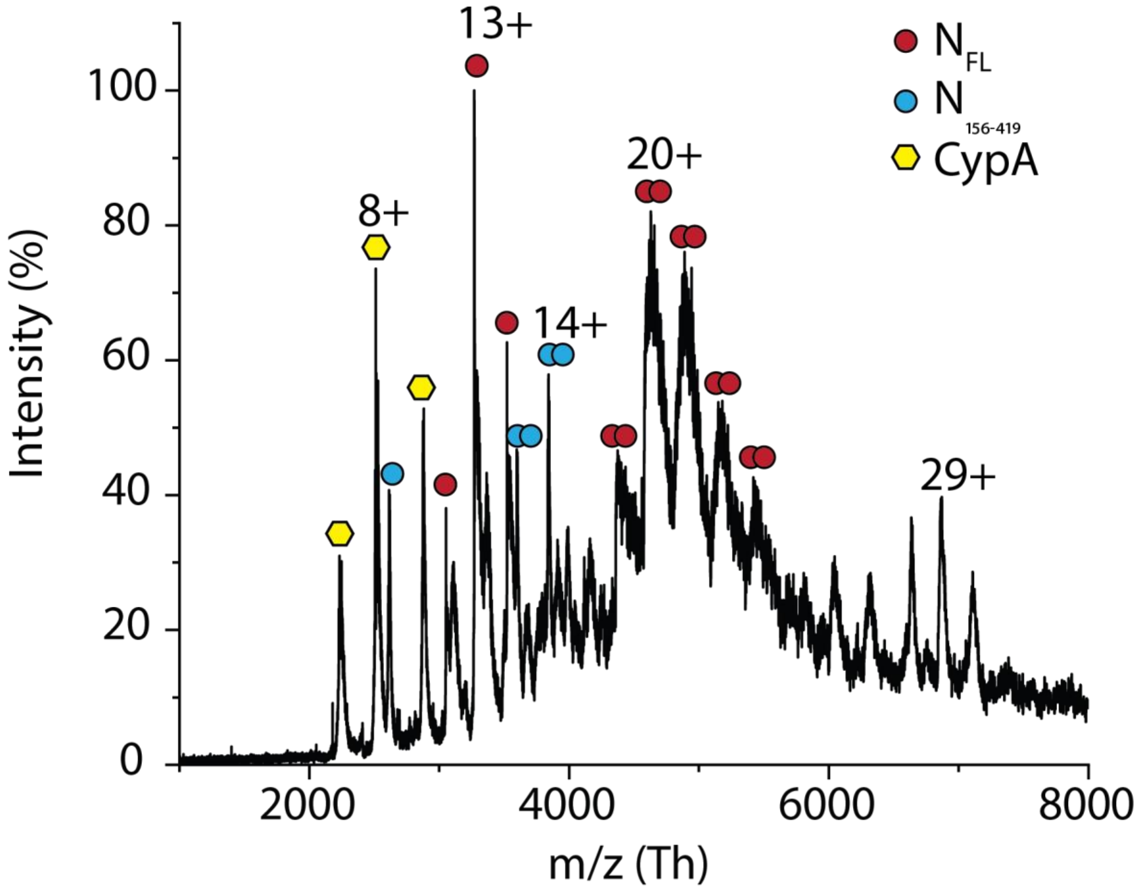

Figure S14. N_{FL} does not interact with cyclophilin A. Mass spectrum shows N_{FL} after incubation with cyclophilin A (CypA) in a 1:1 molar ratio. Charge state distributions are observed for monomeric CypA centered at a charge state of 8+ (yellow hexagons), NFL monomers and dimers centered at 13+ and 20+, respectively (red circles), $N_{156\text{-}419}$ monomers and dimers (blue circles), and a charge state distribution at *m/z* >6000 centered at 29+. No obvious interaction between CypA and N_{FL} is observed.

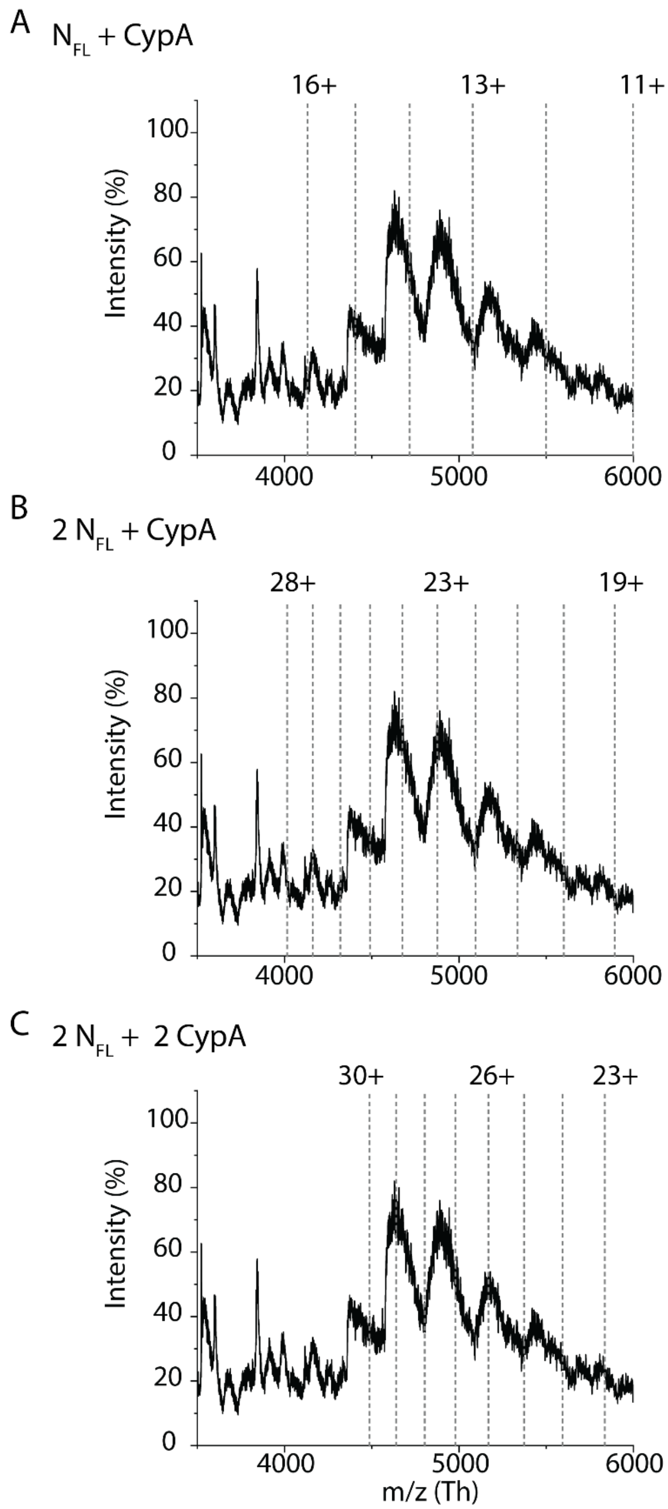

Figure S15. Theoretical peak series for N_{FL}-CypA complex formation overlaid onto the measured mass spectrum for N_{FL} + CypA. The grey dashed lines represent the expected *m/z* values for (A) a complex of N_{FL} bound to CypA, (B) N_{FL} dimers bound to CypA, and (C) N_{FL} dimer bound to CypA dimers. No observed peaks match the anticipated distributions, therefore N_{FL} and CypA do not strongly interact, if at all.

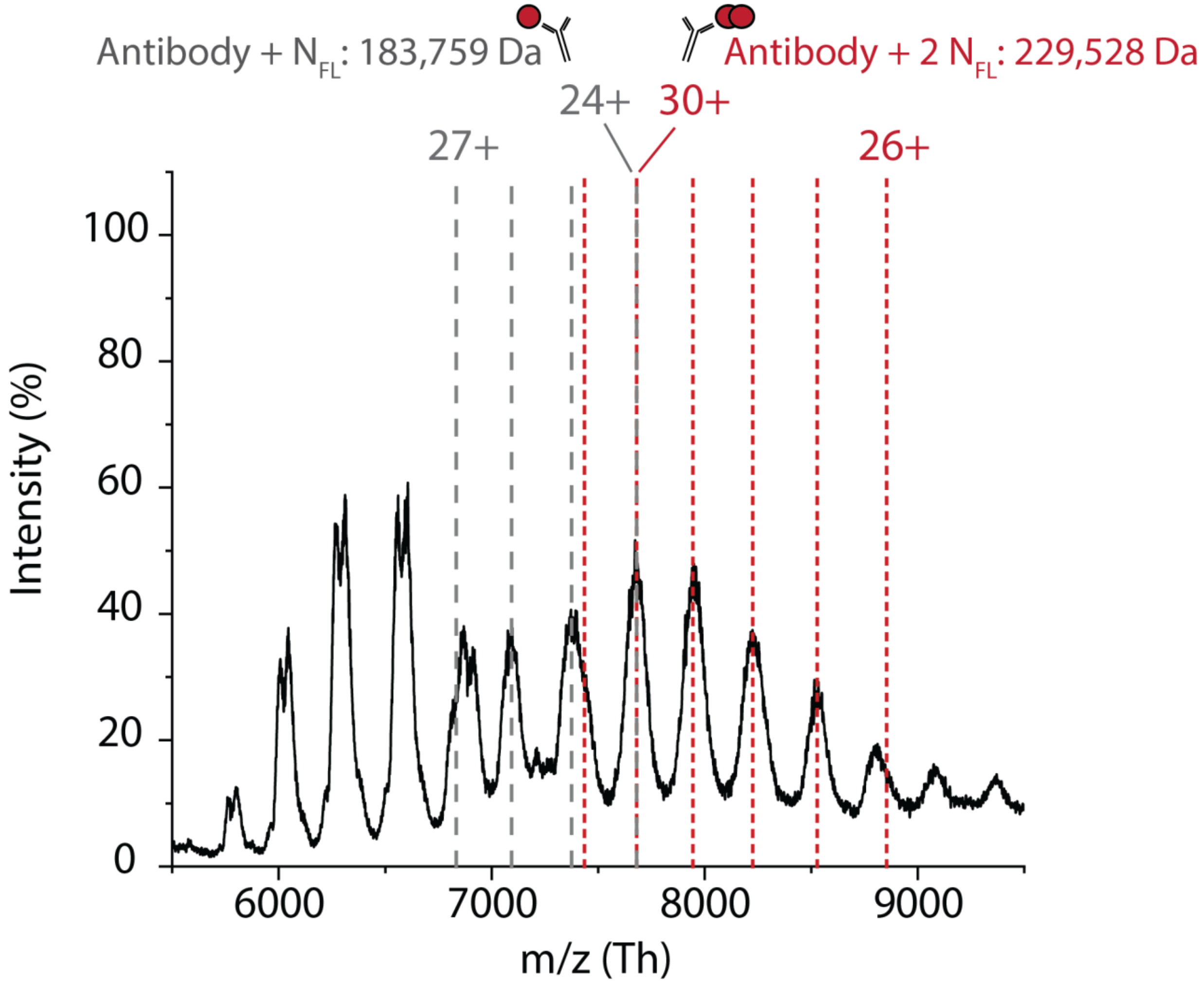

Figure S16. Calculated charge state distributions for one and two N_{FL} bound to a monoclonal antibody. The grey dashed lines represent the *m/z* values for charge states 24+ through 27+ for the antibody bound to one N_{FL} corresponding to a mass of 183,759 Da. The red dashed lines represent the *m/z* values for charge states 26+ through 30+ for the antibody bound to two N_{FL} corresponding to a mass of 229,528 Da. The expected masses were calculated using the lowest deconvoluted mass of the antibody (137,990 Da). The deconvoluted masses for the measured distributions (black trace) are 183,931 ± 102 Da and 229,984 ± 36 Da, respectively.

References

[1] L. M. Smith, N. L. Kelleher. Proteoform: a single term describing protein complexity. *Nat. Methods*. **10**, 186–187 (2013).
